# Robust Concentration and Frequency Control in Oscillatory Homeostats

**DOI:** 10.1371/journal.pone.0107766

**Published:** 2014-09-19

**Authors:** Kristian Thorsen, Oleg Agafonov, Christina H. Selstø, Ingunn W. Jolma, Xiao Y. Ni, Tormod Drengstig, Peter Ruoff

**Affiliations:** 1 Department of Electrical Engineering and Computer Science, University of Stavanger, Stavanger, Norway; 2 Centre for Organelle Research, University of Stavanger, Stavanger, Norway; Morehouse School of Medicine, United States of America

## Abstract

Homeostatic and adaptive control mechanisms are essential for keeping organisms structurally and functionally stable. Integral feedback is a control theoretic concept which has long been known to keep a controlled variable 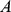 robustly (i.e. perturbation-independent) at a given set-point 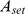 by feeding the integrated error back into the process that generates 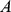. The classical concept of homeostasis as robust regulation within narrow limits is often considered as unsatisfactory and even incompatible with many biological systems which show sustained oscillations, such as circadian rhythms and oscillatory calcium signaling. Nevertheless, there are many similarities between the biological processes which participate in oscillatory mechanisms and classical homeostatic (non-oscillatory) mechanisms. We have investigated whether biological oscillators can show robust homeostatic and adaptive behaviors, and this paper is an attempt to extend the homeostatic concept to include oscillatory conditions. Based on our previously published kinetic conditions on how to generate biochemical models with robust homeostasis we found two properties, which appear to be of general interest concerning oscillatory and homeostatic controlled biological systems. The first one is the ability of these oscillators (“oscillatory homeostats”) to keep the average level of a controlled variable at a defined set-point by involving compensatory changes in frequency and/or amplitude. The second property is the ability to keep the period/frequency of the oscillator tuned within a certain well-defined range. In this paper we highlight mechanisms that lead to these two properties. The biological applications of these findings are discussed using three examples, the homeostatic aspects during oscillatory calcium and p53 signaling, and the involvement of circadian rhythms in homeostatic regulation.

## Introduction

The biological motivation of this work can be summarized as follows: How can homeostatic mechanisms possibly work when many or even most of the regulatory processes within a cell are based on oscillations? Versions of this question and how oscillatory processes participate in homeostatic and adaptive mechanisms have been repeatedly asked and discussed [1]–[5]. Our aim is to identify and build homeostatic/adaptive motifs on a rational basis with possible applications within physiology and synthetic biology. In this paper we apply control-engineering and kinetic methods and show how the classical concept of homeostasis [6], [7] is linked to oscillatory behavior. We demonstrate how biological oscillators can have robust (perturbation-independent) homeostatic/adaptive behaviors both with respect to average concentration of a regulated variable and with respect to a robust control of the oscillator's frequency. By taking three examples, we argue that such properties appear closely linked to the controlled period lengths of the p53-Mdm2 oscillatory system and circadian rhythms [1], [8] or to the homeostatic regulation of cytosolic calcium during signaling [9].

Organisms have developed defending homeostatic mechanisms in order to survive changing or stressful conditions by maintaining their internal physiologies at an approximately constant level [7], [10], [11]. In this respect, many compounds are tightly regulated within certain concentration ranges, because they are essential for cellular function, but may lead to dysfunction and diseases when their concentrations are outside of their regulated regimes. The term “homeostasis” was introduced by Cannon [6], [7] to indicate that the internal milieu of an organism is regulated within narrow limits. The examples Cannon addresses in 1929 [6] are still actual research topics, such as the regulations of body temperature, blood sugar, blood calcium and blood pH levels [12]–[15]. Today many more homeostatic controlled compounds have been identified, including hormones [16], transcription factors and transcription factor related compounds [17], cellular ions such as plant nitrate levels [18], [19], iron [20], and calcium [21]. The Supplementary Material of Ref. [22] contains further examples.

Because many biochemical processes are oscillatory [1], [8], [23]–[27], Cannon's definition of homeostasis has been perceived as unsatisfactory and various alternative homeostasis concepts have been suggested. The term *predictive homeostasis* [1] has been introduced in order to stress the anticipatory homeostatic behavior of circadian regulation. Other concepts include *allostasis* [2], [5] to focus on the concerted and interwoven nature of the defending mechanisms, *rheostasis* [3] to put emphasis on set-point changes, and *homeodynamics* [4] to stress the nonlinear kinetic behaviors of the defending mechanisms as part of an open system.

**Figure 1:**
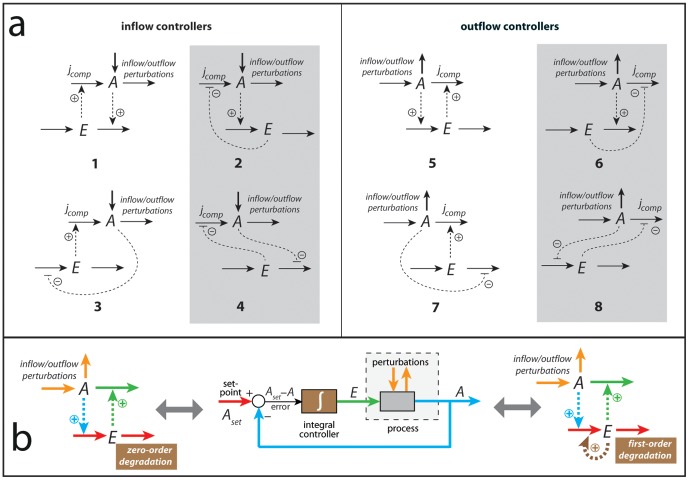
A basic set of two-component homeostatic controller motifs with two implementations of integral control. (a) Compound 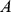 is the homeostatic controlled variable and 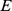 is the controller or manipulated variable [22]. The motifs fall into two classes termed as inflow and outflow controllers, dependent whether their compensatory fluxes 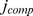 add or remove 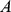 from the system. In motifs outlined in gray the controller compound 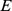 inhibits the compensatory flux, while in the other motifs 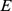 activates the compensatory flux. (b) middle figure shows a standard control engineering flow chart of a negative feedback loop, where the negative feedback results in the subtraction of the concentration of 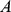 (blue line) from 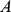
's set-point (red line) leading to the error 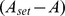. The error feeds into the integral controller (brown box). The controller output (the integrated error) is the concentration of 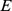 (green line) which regulates the process that creates 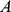. The perturbations which affect the level of 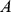 are indicated in orange color. (b) left panel shows the structure of negative feedback (outflow) controller 5. The colors correspond to those of the control engineering flow chart. For example, the set-point (red) is given by the ratio between removing and synthesis rates of 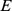, while the integral controller (brown) is related to the processing kinetics of 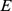, in this case 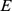 is removed by zero-order [22], [43]. (b) right panel shows the same outflow controller (motif 5). The only difference is that the integral controller is now represented by a first-order autocatalytic formation (indicated by brown dashed arrow) and a first-order removal with respect to 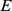 [45].

The appearance of cybernetics together with system theory [28]–[31] caused an interest to understand homeostasis and biological control from the angle of system analysis and control theory [32]–[39] by introducing control-engineering concepts such as *integral control* [22], [40]–[45]. Integral control allows to keep a controlled variable (say *A*) precisely and robustly at a given set-point 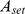 by feeding the integrated error back into the process by which *A* is generated [46]. To gain insights how integral control and homeostasis may appear in biochemical and physiological processes, we started [43] to study two-component negative feedback controllers, where one component is the (homeostatic) controlled variable *A*, while the other is the manipulated or controller variable *E*. Each controller consists of the two species *A* and *E* and three fluxes, the inflow and outflow to and from *E* and an *E* -controlled compensatory flux (either inflow or outflow) of *A*, denoted 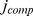. The compensatory flux compensates for disturbances in the level of *A* caused by perturbations in other uncontrolled inflows/outflows of *A*. By considering activating or inhibitory signaling events from *A* to *E* and vice versa, eight basic negative feedback configurations (controller motifs, Fig. 1a) can be created [22], [47]. Two kinetic requirements leading to integral control have so far been identified, one based on a zero-order kinetic removal of the manipulated variable *E* [22], [43], [48], the other on an autocatalytic formation of *E* in association with a first-order degradation [45]. Fig. 1b gives a brief summary of these two kinetic approaches by using motif 5 as an example. For details, the reader is referred to [22], [45]. We feel that this approach provides a rational basis to build networks which allow to view the behaviors of the individual controllers and to understand emergent properties of the overall network. By combining individual controller motifs with integral control we previously showed that an integrative and dynamic approach to cellular homeostasis is possible, which includes storage, excretion and remobilization of the controlled variables [19], [22], [49].

In the present study we extend the concept of homeostasis to include sustained oscillatory or pulsatile conditions. We show that oscillatory homeostats based on the controller motifs in Fig. 1a can maintain robust homeostasis in *A*. For controllers where *E* is inhibiting the compensatory flux (motifs 2, 4, 6, and 8, Fig. 1a), the frequency can be shown to depend on the level of *E* and therefore on the applied perturbation strength. In this class of controllers the frequency generally increases upon increased perturbation strengths; here we use motif 2 as a representative example. For the remaining controller motifs the frequency has been found to be less dependent upon perturbations. As a representative example for this behavior we use motif 5. We further show that robust frequency control can be achieved by either using additional controllers, which keep the average levels of *A* and *E* homeostatic regulated, or by using the intrinsic harmonic/quasi-harmonic properties of motifs 1 or 5. The biological significance of these findings is discussed with respect to the oscillatory signaling of cytosolic calcium and p53, as well as the regulating properties of circadian rhythms with respect to homeostasis and temperature compensation.

## Results

### Kinetic Approach to Implement Integral Control

We consider the negative feedback motifs in Fig. 1. A general condition for integral control can be formulated if the rate equation of the manipulated variable *E* allows for a rearrangement in form of two functions 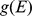 and 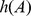, and where the integral of 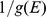 with respect to *E* exists and can be written as 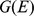. Then, the set-point in *A* is determined by the solution of 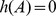, i.e. 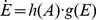
(1)Rearranging Eq. 1 and requiring steady state conditions gives: 
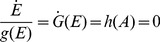
(2)Eq. 2 has been applied for nonoscillatory steady states with 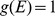 by using zero-order kinetic degradation/inhibition of *E* [22], [43] or with 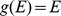 by using first-order autocatalytic formation and degradation in *E* [45]. Other functions of 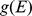 may be possible but plausible reaction kinetic mechanisms need to be identified. For the sake of simplicity, we consider here that integral control is achieved by a zero-order removal of 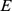 using 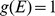.

To extend the condition of Eq. 2 to sustained stable and marginally stable oscillations, we observe that the integral of the periodic reaction rates 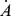 and 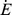 along a closed orbit 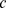 in the system's phase space is zero. For 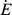 this can be written as: 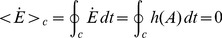
(3)


Dependent on whether *A* is activating or inhibiting the production or removal of *E*, two expressions for the set-point of the oscillatory controller can be derived from Eq. 3. In case *A* is activating (motifs 1, 2, 5, 6) and by assuming first-order kinetics with respect to *A* in the rate equation for *E*, the set-point of *A* is given by (see Eq. S1 in (File S1)) 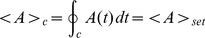
(4)where the integral is taken along one (or multiple) closed and stable orbit(s) in the system's phase space. With increasing time 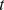, the average concentration of 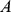, 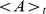, will approach its set-point 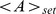, i.e., 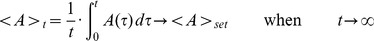
(5)


When 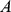 is inhibiting the production or removal of 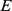 (motifs 3, 4, 7, 8) and assuming (for the sake of simplicity) that the inhibiting term has a first-order cooperativity with respect to 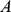 with an inhibition constant 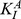, the following expression is conserved and perturbation-independent (see derivation in File S1, Eq. S8): 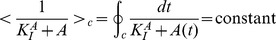
(6)


### Homeostasis by Oscillatory Controllers

To illustrate the homeostatic response of the oscillatory controllers, we use, as mentioned above, conservative and limit-cycle versions of inflow controller motif 2 and outflow controller motif 5 as representative examples. These motifs have been chosen, because they represent different ways to achieve negative feedback and homeostasis of the controlled variable *A*. In motif 2 (as in motifs 4, 6, and 8) *E* inhibits the compensatory flux, while in motif 5 (as in motifs 1, 3, and 7) the compensatory flux is activated by *E*. A limit-cycle version of motif 6 will be used to discuss cytosolic Ca^2+^ oscillations in terms of a homeostatic mechanism.

#### Conservative Oscillatory Controllers

A conservative system is a system for which an energy or Hamiltonian function (*H*-function) can be found and for which the values of *H* remain constant in time. Conservative oscillators show periodic motions characterized by that they in phase space do not occur in isolation (i.e. they are not limit cycles). For a given *H*-level *h* a periodic motion (a closed path in phase space) is surrounded by a continuum of near-by paths, obtained for neighboring values of *h* [50]. The dynamics of a two-component conservative oscillator can be derived from the *H*-function using the following equations: 
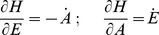
(7)which are analogous to the Hamilton-Jacobi equations from classical mechanics. In general, solutions of these equations are not necessarily oscillatory, but here we focus only on the conservative oscillators, which can be derived from the eight controller motifs (Fig. 1a). Dependent on how integral control is implemented, some of the conservative oscillators are well-known; they are: the *harmonic oscillator* [51] based on either motifs 1 or 5 (using zero-order implementation of integral control; see left panel in Fig. 1b), the *Lotka-Volterra oscillator* [45], [52], [53] also here based on motifs 1 or 5 (but using the autocatalytic implementation of integral control; see right panel in Fig. 1b), and Goodwin's oscillator from 1963 [54] based on motif 2. In the literature the Goodwin oscillator comes in two versions, which are both based on motif 2. There is a conservative oscillator version from 1963 [54] with two components. There is also another version from 1965 with three components [55]. The difference between the two versions lies in the kinetics of the degradation rates of the oscillators' components. In the 1965 three-component version the degradation rates are first-order with respect to the degrading species, while in the conservative case (1963 version) the degradation rates have zero-order kinetics. These kinetic differences change the oscillatory behavior of the two systems significantly. To get limit-cycle oscillations, it is well-known from the literature [56] that the three-dimensional system where the components are degraded by first-order kinetics requires a cooperativity of the inhibiting species of about 9 or higher. Our results presented here using motif 2 confirms Goodwin's 1963 results that when components are degraded by zero-order kinetics the system can oscillate with a cooperativity of 1 with respect to the inhibiting species *E*. Here we also extend Goodwin's results by showing that *limit-cycle oscillations* can be created based on motif 2, but still using a cooperativity of 1 with respect to the inhibiting species *E* (see below).

The following two requirements are needed to get conservative oscillations for any motif from Fig. 1a: (i) integral control has to be implemented in the rate equation for *E*, and (ii) all removal of *A* should either occur by zero-order kinetics with respect to *A*, or, when the removal of 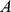 is first (or nth)-order with respect to *A*, the formation of *A* needs to be a first (or nth)-order autocatalytic reaction [45]. When conditions (i) and (ii) are fulfilled, a function 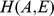 can be constructed, which describes the dynamics of the system analogous to the Hamilton-Jacobi equations from classical mechanics, where the form of *H* depends on the system's kinetics. Details on how *H* is constructed for the various situations is given in File S1.

**Figure 2:**
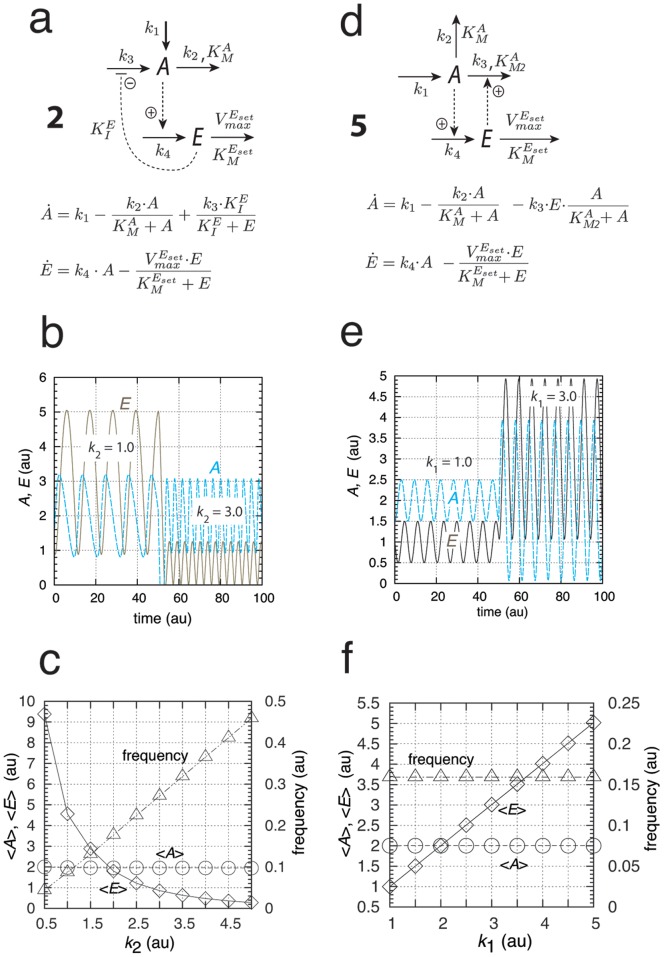
Representation and kinetics of conservative oscillators based on motif 2 and motif 5. (a)–(c) “Goodwin's oscillator” (motif 2). Conservative oscillations occur when 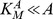 and 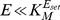; the latter condition introduces integral feedback and thereby robust homeostasis [22], [43]. (b) Conservative oscillations in 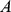 and 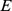, with 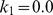, 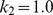, 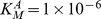, 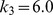, 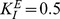, 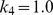, 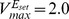, 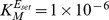. Initial concentrations: 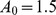, 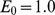. At time t = 50.0 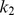 is changed from 1.0 to 3.0. (c) 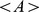, 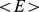, and frequency as a function of the perturbation 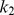. While the frequency increases and 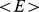 decreases with increasing 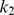, 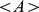 is kept at its set-point 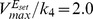. (d)–(f) Harmonic oscillator representation of motif 5. Conservative (harmonic) oscillations occur when 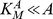 (or 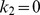) and 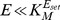. (e) Harmonic oscillations in 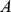 and 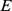, with 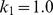 (the perturbation), 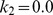, 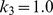, 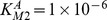, 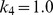, 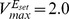, and 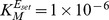. At time t = 50.0 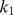 is changed from 1.0 to 3.0. Initial concentrations: 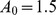, 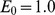. (f) 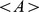, 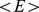, and frequency as a function of the perturbation 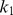. Typical for the harmonic oscillator is the constancy of the frequency upon changing 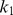 values. 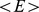 increases with increasing 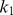, while 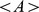 is kept at its set-point 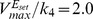.

Fig. 2a shows a reaction kinetic representation of motif 2, which is closely related to Goodwin's 1963 oscillator [54]. It was Goodwin who first drew attention to the analogy between the dynamics of a set of two-component cellular negative feedback oscillators and classical mechanics [54]. In this inflow-type of controller, increased outflow perturbations (i.e., increased 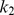 values) are compensated by a decreased average amount of 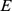 (i.e., 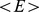, Fig. 2b), thereby neutralizing the increased removal of 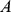 by use of an increased compensating flux 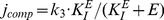
(8)


In this way the average level of 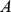, 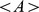, is kept at its set-point 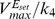 (see Eq. S5 in the File S1). During the adaptation in 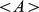 (when 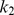 is changed) the controller's frequency as well as the 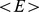-level are affected. The frequency 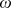 for each of the eight conservative oscillators can roughly be estimated by a harmonic approximation (see File S1), which in case of motif 2 (Fig. 2a) is given by (assuming 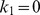) 
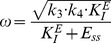
(9)

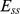
 (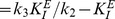) is the steady state of 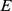, which is obtained when 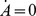 (Fig. 2a). Because the level of 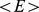 is decreasing with increasing 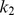 values, Eq. 9 indicates, and as shown by the computations in Figs. 2b and 2c, that the frequency of the oscillator increases with increasing perturbation strengths (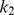 values) while keeping 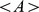 at its set-point. In fact, the increase in frequency upon increased perturbation strengths appears to be a general property of oscillatory homeostats, where the manipulated variable 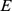 inhibits the compensatory flux (for limit-cycle examples, see below).

At high 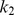 values, i.e., when the 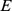 level becomes lower than 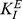, the compensatory flux 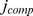 approaches its maximum value 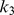. At this stage the homeostatic capacity of the controller is reached. Any further increase of 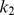 cannot be met by an increased compensatory flux and will therefore lead to a breakdown of the controller. For discussions about controller breakdowns and controller accuracies, see Refs. [22], [48].

The scheme in Fig. 2d shows outflow controller motif 5, which will compensate any inflow perturbations of 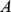 (due to changes in 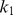) by increasing the compensatory flux 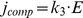
(10)


When 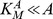 and 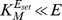 the oscillator is harmonic and is described by a single sine function which oscillates around the set-point 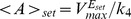 with frequency 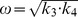 and a period of 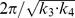. Increased levels in 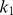 (Figs. 2e and 2f) are compensated by increased 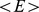 levels which keep 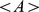 at its set-point. Harmonic oscillations can also be obtained for the counterpart inflow motif 1 (see Fig. S9 and Eqs. S44–S50 in File S1).

For the harmonic oscillators (motifs 1 or 5) 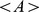-homeostasis is kept by an increase in 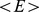, which matches precisely the increase in the (average) compensatory flux without any need to change the frequency. For the other motifs either an increase or a decrease in frequency is observed with increasing perturbation strengths dependent whether 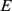 inhibits or activates the compensatory flux, respectively.

#### Limit-Cycle Controllers

**Figure 3:**
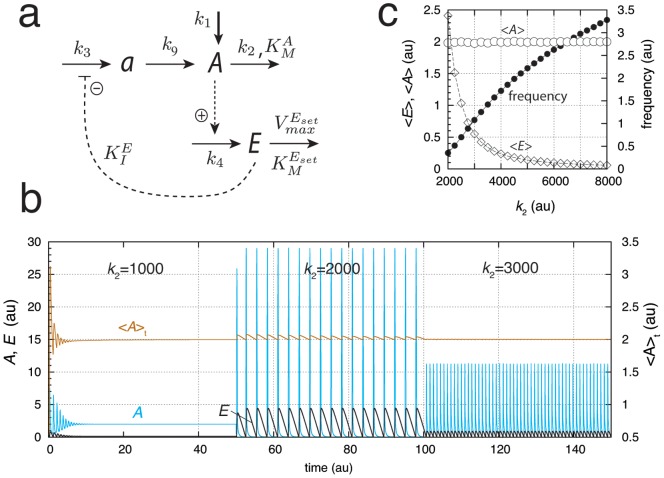
A limit-cycle model of controller motif 2. (a) Reaction scheme. Rate equations: 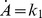 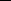 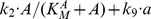; 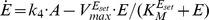; 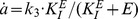 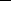 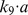. (b) Homeostatic response of the model for three different perturbations (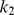 values). For time 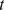 between 0 and 50 units, 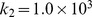, for 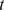 between 50 and 100 units, 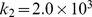, and for 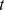 between 100 and 150 units, 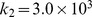. In the oscillatory case 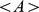 at time 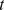 is given as 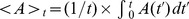 (ordinate to the right) showing that 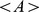 is under homeostatic control despite the fact that 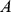 peak values may be over one order of magnitude larger than the set-point. (c) 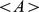, 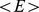, and frequency values as a function of 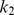. Simulation time for each data point is 100.0 time units. Note that 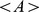 is kept at 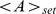 independent of 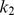. Rate constant values (in au): 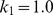, 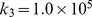, 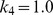, 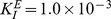, 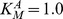, 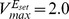, 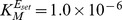, and 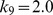. It may further be noted that the degradation kinetics with respect to 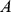 are no longer zero-order as required in the conservative case (Figs. 2a–c). Initial concentrations in (b): 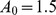, 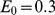, and 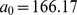. Initial concentrations in (c) for each data point: 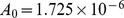, 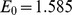, and 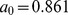.

The conservative oscillatory controllers described above can be transformed into limit-cycle oscillators by including an additional intermediate, and, as long as integral control is present, homeostasis in 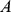 is maintained by means of Eq. 4 or 6. Fig. 3a gives an example of a limit-cycle homeostat using motif 2. Dependent on the rate constants the oscillations can show pulsatile/excitable behavior (Fig. 3b). In these pulsatile and highly nonlinear oscillations 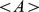 homeostasis is maintained at the set-point 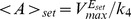, although the peak value in 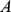 exceeds the set-point by over one order of magnitude (Fig. 3b). As already observed for the conservative case, an increase in the perturbation strength (i.e., by increasing 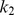) leads to an increase in frequency while homeostasis in 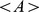 is preserved (Fig. 3c).

**Figure 4:**
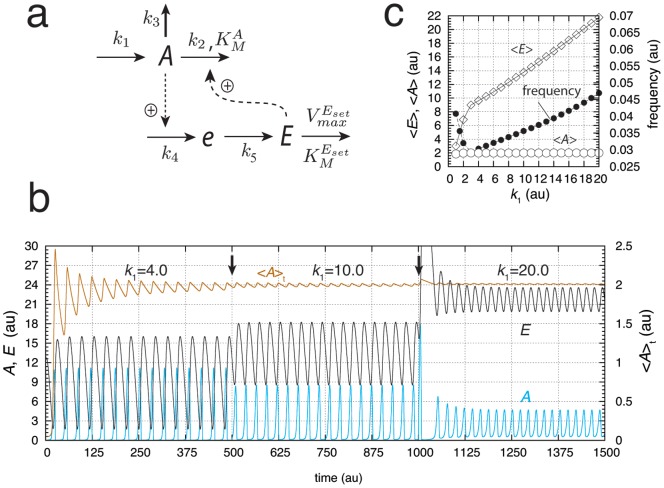
A limit-cycle model of controller motif 5. **(a).** Rate equations: 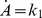 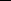 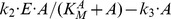; 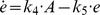; 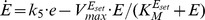. (b) Homeostatic behavior in 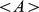 illustrated by three different perturbations (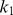 values). At time 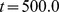 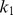 is changed from 4.0 to 10.0, and at 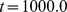 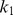 is changed from 10.0 to 20.0 (indicated by solid arrows). The set-point of 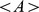 is given as 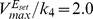. Rate constant values: 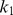 is variable, 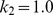, 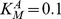, 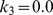, 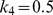, 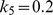, 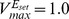, and 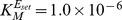. Initial concentrations: 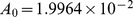, 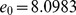, and 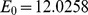. (c) 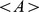, 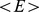, and frequency values as a function of 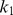 showing that 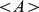 is kept at the set-point independent of 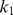. Rate constants as in (b). Initial concentrations for each data point: 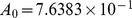, 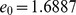, and 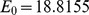. Simulation time for each data point is 10000.0 time units.

Similarly, a limit-cycle homeostat of motif 5 can be created (Fig. 4a) by including intermediate 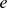 and maintaining integral control with respect to 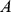. With increasing perturbation strengths (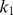 values, Fig. 4b), homeostasis in 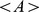 is maintained by increasing 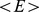. Compared to the conservative situation (Fig. 2f), the frequency now shows both slight decreasing and increasing values. However, the overall frequency changes are not as large as for motif 2, indicating that similar to the harmonic case, the frequency of the motif 5 based oscillator has a certain intrinsic frequency compensation on 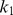-induced perturbations (Fig. 4c).

### Robust Frequency Control and Quenching of Oscillations

In this section we present for the first time biochemical models that can show robust (perturbation-independent) frequency control. There are several biological oscillators where the frequency/period is under homeostatic regulation. Probably the best known example is the temperature compensation of the circadian period, i.e. these rhythms show an approximately constant period length of about 24 h at different but constant temperatures [57]. Temperature compensation is also observed in certain ultradian rhythms [58], [59]. Another biological oscillator with a fairly constant period is the p53-Mdm2 system [60], where the number of oscillations may indicate the strength of the DNA damage in the cell [61].

We show two ways how robust frequency control can be achieved. One is due to the presence of quasi-harmonic kinetics, i.e. the system, although still being a limit-cycle oscillator, behaves more like a harmonic oscillator. On basis of experimental results, we believe that the p53-Mdm2 system falls into this category (see discussion below). In the other approach, frequency homeostasis is obtained by regulating 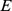 itself by additional inflow/outflow controllers 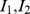. This approach leads to many possible ways how 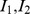 can interact with the central negative feedback 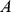-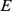 loop/oscillator and several ways are illustrated using motif 2 and motif 5. Such an approach may apply to the period homeostasis of circadian rhythms (see discussion below).

#### Robust Frequency Control by Quasi-Harmonic Kinetics

**Figure 5:**
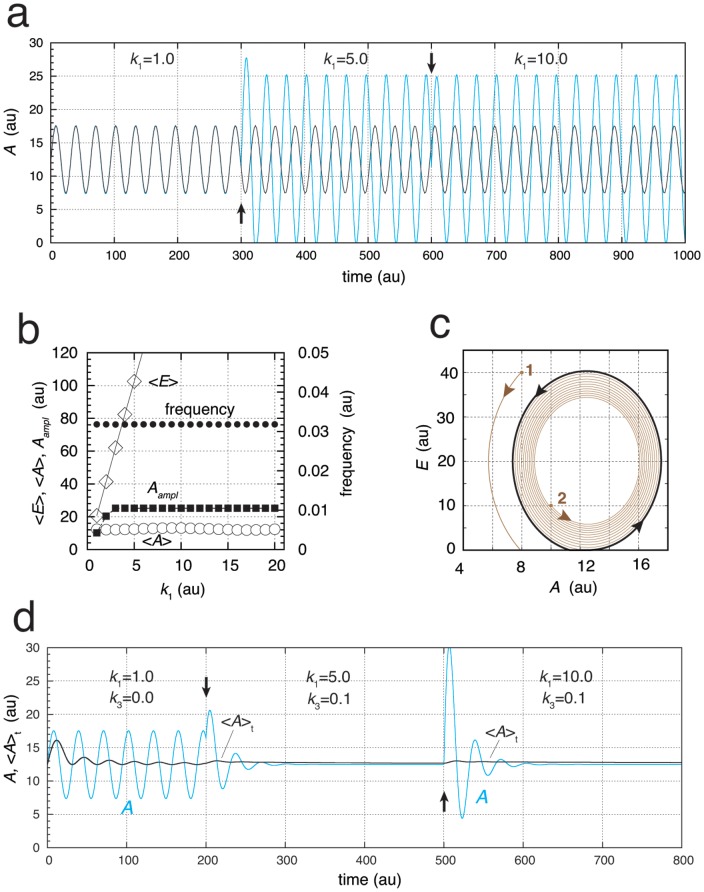
Quasi-harmonic behavior of motif 5 oscillator (Fig. 4a). For time 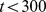, a perfect overlay between the numerical calculation of 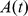 (blue color) and the single harmonic 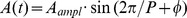 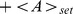 (black color) is found, where 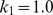, 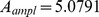, 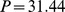, 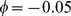, and 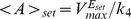 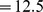. 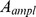 and 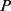 represent the numerically calculated amplitude and period length, respectively. 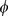 was adjusted to give a closely matching overlay. Other rate constant values (numerical calculations): 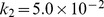, 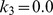, 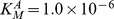, 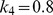, 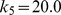, 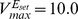, and 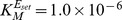. Initial concentrations: 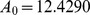, 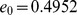, and 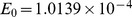. At times 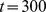 and 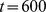 (solid arrows) 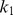 is changed to respectively 5.0 and 10.0. For these 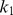 values the amplitude of 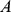 has reached its maximum, which is twice the value of the set-point. (b) 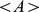, 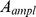, 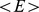, and frequency as a function of 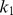. Simulation time for each data point is 1000.0 time units. (c) Demonstration of limit-cycle behavior of the quasi-harmonic oscillations. Same initial conditions as in (a) with 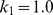, and 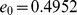. (d) Same system as in (a), but at times 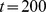 and 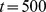 (solid arrows) 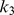 is changed and kept to 0.1. The oscillations are efficiently quenched, but 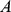 remains under homeostatic control.

We consider now the case when the intermediate that has been implemented to obtain limit-cycle behavior (compounds 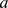 or 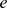 in Figs. 3a or 4a) obeys *approximately* the steady-state assumption, i.e., 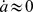 or 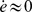. We term the oscillators' resulting behavior as *quasi-conservative*, because these systems still have a limit-cycle, but behave also as a conservative system. An interesting case occurs when the system is quasi-harmonic, i.e. when motifs 1 or 5 are used. In this case the limit-cycle oscillations and the frequency can approximately be described by a harmonic oscillator, i.e., a single sine function. This is illustrated in Fig. 5 where an increased 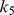 value is applied to the scheme of Fig. 4a (which leads to 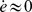). Fig. 5a shows the oscillations for three different perturbations (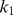 values). The oscillations in 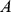 show a practically perfect overlay with a single sine function, outlined in black for 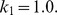 When 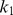 is increased the oscillations (outlined in blue) undergo a phase shift and an increase in amplitude, but the frequency stays constant at the value of the (quasi) harmonic oscillator. For high 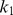 values the 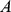-amplitude of the oscillator becomes saturated, which is a secondary effect of the oscillator's homeostatic property. Due to symmetry reasons and because the oscillator is locked on to the harmonic frequency, the value of 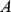 cannot exceed beyond twice the level of its set-point, which in this case has been set to 12.5 (Figs. 5a and 5b). As in the harmonic case (Fig. 2f), 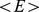 increases with increasing 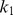 (Fig. 5b). Fig. 5c shows the approach to the limit-cycle (outlined in black). When 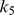 increases further and the steady state approximation for 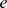 becomes better and better, the limit-cycle disappears and the system becomes purely harmonic.

#### Quenching of Oscillations in Quasi-Conservative Systems

A requirement to obtain conservative oscillations and an oscillatory promoting condition for limit cycle oscillations is the presence of zero-order degradation in 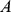. Changing the zero-order degradation in 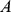 may lead to the loss of oscillations. For example, in quasi-conservative systems the oscillations can be effectively quenched by either adding a first-order removal term with respect to 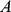 (with rate constant 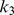, Fig. 4a) or by replacing the zero-order kinetics degradation in 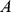 (using 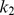, 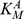) by first-order kinetics with respect to 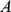, or by increasing 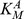. Fig. 5d illustrates the suppression of the quasi-harmonic oscillations by adding a first-order removal with respect to 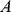. In contrast, when an oscillatory system does not show quasi-conservative kinetics, addition of a first-order removal with respect to 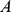 does not necessarily abolish the oscillations. A detailed parameter analysis showing how the value of 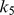 affects the period of the oscillations and how first-order degradation in 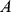 affects the size of the parameter space in which sustained oscillations are found is given in (Figs. S10 and S11 in File S1).

#### Robust Frequency Homeostasis by Control of 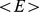


When considering the relationship between 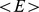 and the frequency, as for example shown in Fig. 3c, we wondered whether it would be possible to design an oscillator with a robust frequency homeostasis by using an additional control of 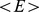. For this purpose, two extra controllers 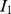 and 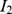 with their own set-points for 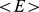 are introduced. Note, that the integral control for 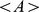 by 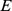 is still operative and has its own defined set-point. In the following we show three examples of robust frequency control using motifs 2 and 5. Two of the examples illustrate different feedback arrangements of 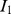 and 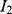 using motif 2. An example using still another arrangement using motif 2 is described in File S1 (Figs. S12–S14).

**Figure 6:**
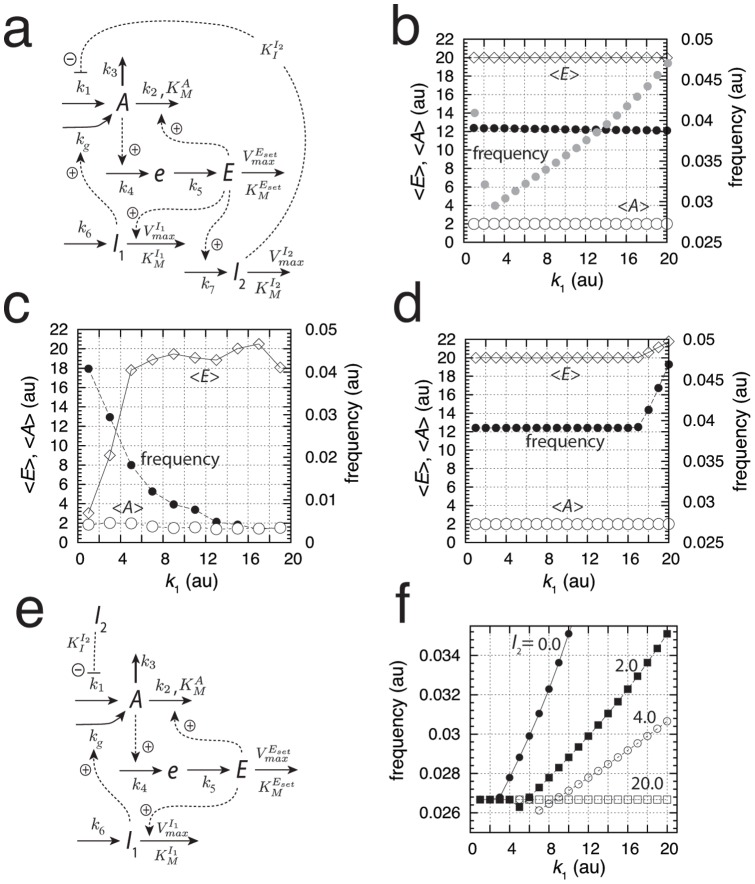
Oscillator based on motif 5 with robust frequency control. (a) Reaction scheme. Rate equations: 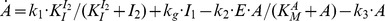; 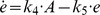; 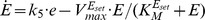; 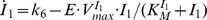; 
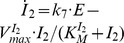
. (b) Demonstration of robust frequency control. 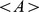, 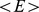, and frequency are shown as functions of 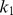. Rate constants: 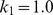, 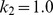, 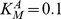, 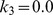, 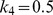, 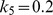, 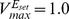, 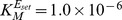, 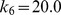, 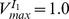, 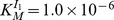, 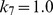, 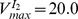, and 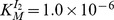. Set-points for 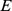 by controllers  and 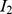 are given as 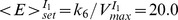 and 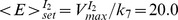, respectively. Initial concentrations for each data point (black dots): 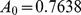, 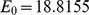, 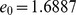, 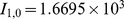, and 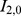 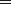 2.7657 × 10^2^. Gray dots show the frequency as a function of 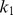 without control by 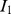 and 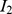. (c) System as in (b), but controller 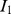 not present. (d) System as in (b), but controller 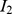 not present. (e) Reaction scheme of oscillator, but with a constant 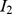 concentration. Rate constants otherwise as in (b). (f) Frequency as a function of 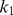 for the system described in (e) using different constant 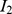 concentrations (indicated within the graph). The homeostatic region of the frequency increases with increasing 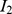 concentrations.

In Fig. 6a a set-up for robust frequency homeostasis is shown by using a limit-cycle oscillator based on controller motif 5. The set-points for 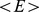, given by the rate equations for 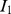 and 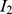, are 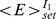 = 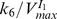 and 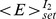 = 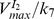. Fig. 6b shows the results for a set of calculations when 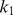 varies from 1 to 20 au. In these calculations it was assumed that the 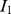 and 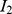 controllers have the same set-point of 20.0 au. In the absence of controllers 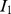 and 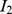, the frequency varies as indicated in Fig. 4c, which in Fig. 6b is shown as gray dots. When 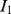 and 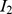 controllers are both active, 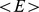 shows robust homeostasis at 20.0 (Fig. 6b) and the frequency is practically constant (black dots). Fig. 6c shows the response when controller 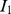 has been “knocked out”. While in this case the 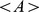 values are still under homeostatic control, 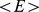 approaches its set-point (defined by 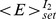) only at high 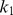 values, but without a control of the frequency. When controller 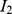 is knocked-out (Fig. 6d), control of 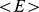 and frequency homeostasis is observed. Due to the absence of controller 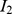, homeostasis in 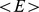 and in the frequency is lost for higher 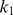 values. The role of 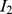 in this type of regulator is to diminish/suppress the inflow to 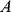 by 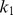, such that controller 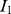 can supply the necessary amount of 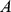 in order to keep 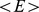 and the frequency under homeostatic control. This mechanism is illustrated in Fig. 6e by a “static” work mode of 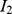, where the concentration of 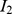 is kept constant. In this case the 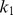-region of frequency homeostasis increases with increasing but constant concentrations of 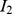 (Fig. 6f).

**Figure 7:**
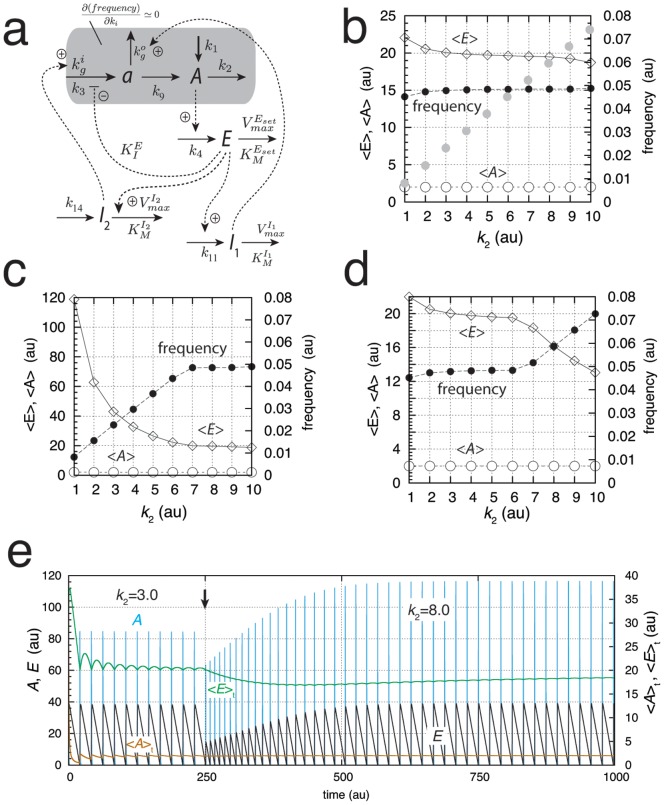
Oscillator based on motif 2 with robust frequency control. (a) Reaction scheme. Rate equations: 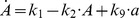; 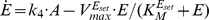; 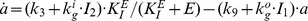; 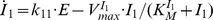; 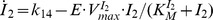. Shaded area indicates part of the model for which the control coefficents of the frequency/period with respect to the parameters within this area become zero when frequency homeostasis is enforced by controllers 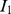 and 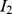. (b) Demonstration of frequency homeostasis by varying 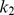. Black dots show the frequency when controllers 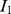 and 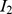 are active. Rate constants: 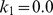, 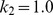, 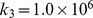, 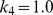, 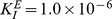, 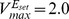, 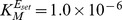, 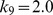, 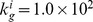, 
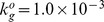
, 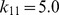, 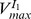 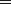 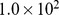, 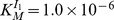, 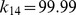, 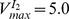, and 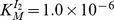. Set-points for 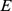 by controllers 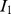 and 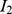 are given as 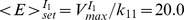 and 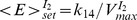 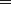 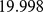, respectively. The set-point 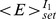 of the *outflow* controller 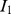 has been set slightly higher than 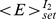 for the *inflow* controller 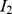 to avoid integral windup and that the controllers work “against” each other [22]. Initial concentrations for each data point (black dots): 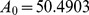, 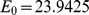, 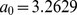, 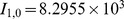, and 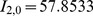. Gray dots show the frequency as a function of 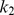 for the uncontrolled case, i.e., in the absence of controllers 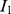 and 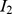. (c) System as in (b), but controller 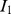 is “knocked out” by setting 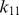 and 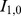 to zero. Homeostasis occurs only at high 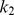 values when controller 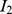 is active. (d) System as in (b), but inflow controller 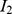 is inactivated by setting 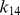 and 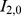 to zero. Frequency homeostasis is observed for low 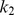 when controller 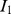 is active. At high 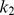 values the frequency homeostasis breaks down, because controller 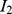 is not present to compensate the increased outflow of 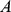, which leads to low 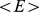 values. (e) Oscillations of system in (b) illustrating frequency homeostasis. At time 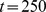 (solid arrow) 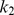 is changed from 3.0 to 8.0. Initial concentrations: 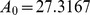, 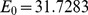, 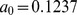, 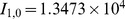, and 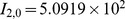.

A corresponding approach to achieve robust frequency homeostasis by using motif 2 is shown in Fig. 7a. The set-up differs from that used for motif 5 (Fig. 6a) by allowing that 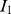 and 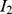 act upon 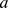 and upstreams of 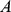. For the sake of simplicity, both controllers are assumed to have set-points at 20.0 au. Note that in this version of the motif 2 oscillator, the removal of 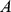 is now purely first-order with respect to 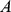 (using only 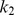). Because motif 2 has been the core for many circadian rhythm models, we will below discuss implications of robust frequency control with respect to properties of circadian rhythms. In this context we note that the region outlined in gray in Fig. 7a shows the part of the oscillator where rate constants have no influence on the frequency, i.e. the sensitivity coefficients 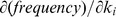 are zero.

Fig. 7b shows the homeostatic behavior in frequency (black dots) in comparison with the uncontrolled oscillator (gray dots). In the controlled case, both 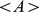 and 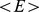 are under homeostatic regulation with set-points of 2.0 au and 20.0 au, respectively. To elucidate the effect of the added controllers 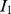 and 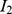, we removed them one by one (knocking them out). In Figs. 7c and 7d controllers 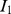 and 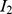 have been removed, respectively. When outflow controller 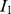 is not operative, the system is not able to remove sufficient 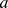 at low 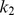 values. In this case 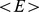 levels are high and unregulated at low 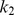
's and showing an increase in frequency. Only at sufficiently high 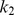 values controller 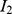 is able to compensate for the decreased levels in 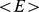. The situation is reversed in Fig. 7d, when controller 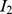 is not operative. At low 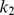 values controller 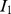 can remove excess of 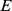 by diminishing the level of 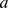 and keeping 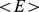 at its set-point. However, the 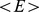 regulation breaks down at high values of 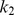, because no additional supply for 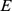 via 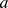 can now be provided. In this way controllers 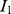/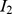 act as an antagonistic pair of outflow/inflow controllers, respectively. Note that the by 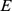 controlled level of 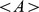 (with set-point of 2.0 au) is kept at its set-point independently whether 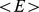 is regulated by 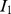/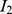 or not. Fig. 7e shows the oscillations when both 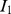 and 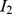 are operative (Fig. 7b, black dots) and 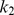 being changed from 3.0 to 8.0 at t = 250.0 units (indicated by arrow). The level of 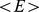 is controlled to its set-point (20.0), while the amplitude of 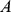 has increased with the increase of 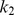. For each spike (after steady state has been established) the average amount of 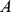 is the same and independent of the value of 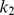, leading to the same frequency and homeostasis in 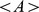.

### Oscillator with Two Homeostatic Frequency Domains

**Figure 8:**
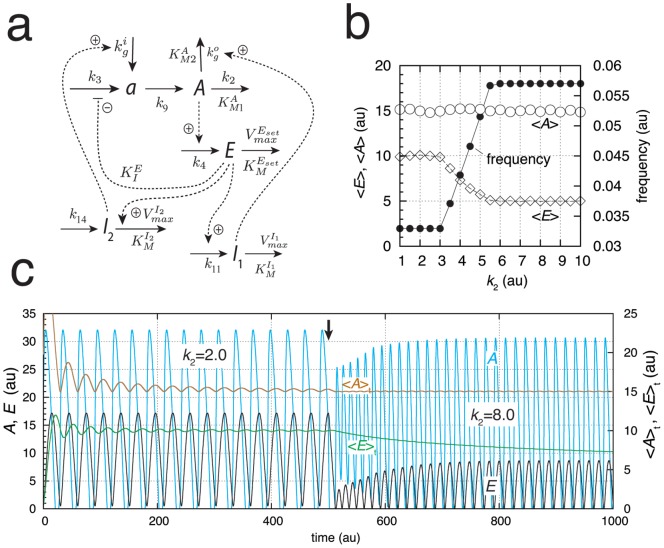
Oscillator based on motif 2 with robust frequency control but alternative feedback regulation by 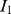 and 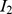. (a) Reaction scheme. Rate equations: 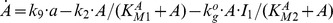, 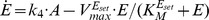; 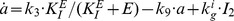; 
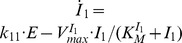
; 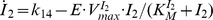. (b) Using different set-points 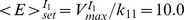 and 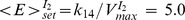, the frequency (solid dots) can switch between two homeostatic frequency regimes, dependent whether 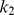 is low or high. The two regimes are separated by a transition zone. Rate constants: 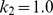, 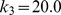, 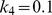, 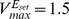, 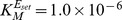, 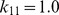, 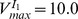, 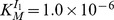, 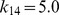, 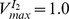, 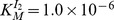, 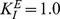, 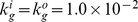, 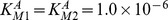. Initial concentrations: 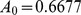, 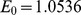, 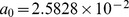, 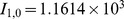, and 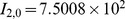. (c) Oscillations of system in (b) illustrating frequency switch. At time 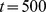 (solid arrow) 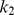 is changed from 2.0 to 8.0. Initial concentrations: 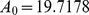, 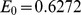, 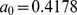, 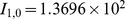, and 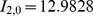.

In the 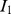 and 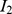-controlled oscillators described above the set-point of 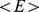 will determine the frequency. Fig. 8a shows an example of a motif-2-based homeostat, where 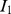 and 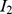 feed back to 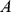 and 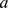, respectively. For an example where 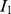 and 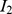 feed back to 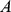 only, see Fig. S12 in File S1. In the calculations of Fig. 8, different set-points for 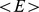 by controllers 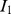 and 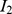 have been chosen. As a result, dependent whether the perturbation strength (value of 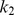) is high or low, the oscillator shifts between two different homeostatic controlled frequency regimes separated by a transition zone (Fig. 8b). Fig. 8c shows the oscillations, 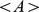 and 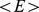 values and the frequency switch when 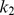 is changed from 3.0 to 8.0.

## Discussion

### Classifications of Biochemical Oscillators and Influence of Positive Feedback

**Figure 9:**
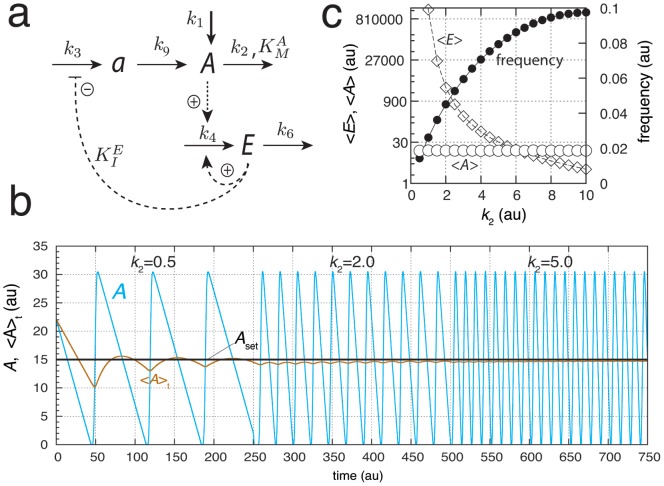
A limit-cycle model of controller motif 2 using autocatalysis as an integral controller. (a) Reaction scheme. Rate equations: 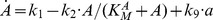; 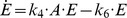; 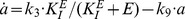. (b) Homeostatic response of the model for three different perturbations (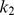 values). For time 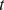 between 0 and 250 units, 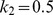, for 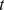 between 250 and 500 units, 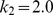, and for 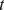 between 500 and 750 units, 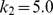. 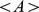 at time 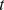 is defined as in Fig. 3. (c) 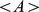, 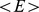, and frequency values as a function of 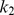. Simulation time for each data point is 2000.0 time units. Note that 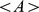 is kept at 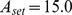 (solid black line) independent of 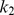. Rate constant values (in au): 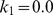, 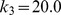, 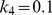, 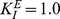, 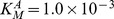, 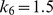, and 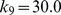. Initial concentrations in (b): 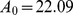, 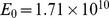, and 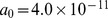. Initial concentrations in (c) for each data point are the same as in (b).

There has been several approaches how chemical and biochemical oscillators can be understood and classified [62]–[66]. The controller motifs shown in Fig. 1a can be considered as a basic set of negative feedback oscillators. For example, the Lotka-Volterra oscillator can be viewed as a negative feedback oscillator based on motifs 1 or 5, but where integral control is implemented in terms of autocatalysis [45] and where the controlled variable 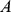 is formed by autocatalysis and degraded by a first-order process with respect to 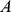. The same motif can show harmonic oscillations, when integral control and removal of the controlled variable is incorporated by means of zero-order kinetics. Two additional oscillator types based on the same motif can be created by implementing mixed autocatalytic/zero-order kinetics for integral control and for the generation/degradation of the controlled variable (‘Text S1’). The other motifs can be extended in a similar way, giving rise to 32 basic (mostly unexplored) oscillator types. This type of classification supplements the one given earlier by Franck, where the eight negative feedback loops where combined with their positive counterparts to create what Franck termed *antagonistic feedback* [63] An often discussed question is the role positive feedback, or autocatalysis, may play in biological oscillators. Using a Monte-Carlo approach Tsai et al. [26] studied the robustness and frequency responses of oscillators with only negative feedback loops and oscillators with a combined positive-plus-negative feedback design. The authors concluded that the combination of a negative and a positive feedback is the best option for having robust and tunable oscillations. In particular, the positive loop appears necessary to make the oscillator tunable at a constant amplitude. We here have shown how homeostasis and tunable oscillators may be achieved without any positive feedback (but generally associated with a changing amplitude). To put our results in relation to those from Tsai et al. [26], we wondered, triggered by the comments from a reviewer, how an oscillator with an autocatalytic-based integral controller might behave in comparison. For this purpose we used controller motif 2 (Fig. 9a), analogous to the scheme shown in Fig. 3a. Interestingly, and in agreement with the findings by Tsai et al. [26], the autocatalytic step resulted now in relaxation-type of oscillations. As expected, the frequency of the oscillator increases with increasing perturbation strengths 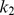, and 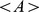 is under homeostatic control (Fig. 9b). However, as indicated by the results of Tsai et al. the oscillator's amplitude has now become independent of 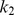
! These results show that Franck's original concept of antagonistic feedback, i.e. combining positive and negative feedback loops in various ways [63] appear to be of relevance for many biological oscillators [26].

### Homeostatic Regulation under Oscillatory Conditions

In his definition of homeostasis Cannon introduced the term *homeo* instead of *homo* to indicate that certain variations in the concentrations of the homeostatic controlled species are still allowed, but within certain limits [6]. As typical examples, Cannon mentions the variations of body temperature, variations in blood sugar, blood calcium, and blood pH levels [6]. We have shown that the concept of homeostasis can be extended to oscillatory conditions and that the term *set-point* still can be given a precise meaning, even when peak values of the controlled variable may exceed the set-point by over one order of magnitude (Figs. 3 and 7). In these cases the set-point relates to the mean value of the oscillatory species, 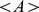. Many compounds are known to be under a tight homeostatic regulation to avoid cellular dysfunction, such as is the case for cytosolic calcium. There is no particular reason to assume that protective homeostatic mechanisms should cease to exist once a compound becomes oscillatory and functions, as in case of calcium, as a signaling device. Allowing a species (such as cytosolic calcium) to oscillate while defending the mean value of these oscillations makes it possible to relay signaling without exposing the cell to long term overload. In the following we discuss three examples where oscillatory homeostats appear to be involved: in the homeostatic regulation of calcium and p53 during oscillations/signaling, and in the homeostatic function and period regulation of circadian rhythms.

#### Calcium Signaling

Cytosolic calcium (Ca^2+^) levels are under homeostatic control to concentrations at about 100 nM while extracellular levels are in the order of 1 mM. High Ca^2+^ concentrations are also found in the endoplasmatic reticulum (ER) and in mitochondria (between 0.1–10 mM), which act as calcium stores. To keep cytosolic Ca^2+^ concentrations at such a low level Ca^2+^ is actively pumped out from the cytoplasm into the extracellular space and into organelles by means of various Ca^2+^ ATPases located in the plasma membrane (PMCA pumps) and in organelle membranes [21], [67]. Dysfunction of these pumps leads to a variety of diseases including cancer, hypertension, cardiac problems, and neurodegeneration [68]–[70]. During Ca^2+^ signaling [71], [72] cytosolic Ca^2+^ levels show oscillations [73]–[75] but signaling can also occur as individual sparks or spikes [76]. Ca^2+^ oscillations have been found to occur in many cell types and differ considerably in their shapes and time scales with peak levels up to one order of magnitude higher than resting levels. Similar to the behavior of stimulated (perturbed) oscillatory homeostats as for example shown in Fig. 3b, Ca^2+^ oscillations have been found to increase their frequency upon increased stimulation of cells [73]–[75]. The frequency modulation of Ca^2+^ oscillations [77] is considered to be an important property for controlling biological processes [75]. The tight homeostatic regulation of cytosolic calcium combined with its oscillatory signaling suggests that oscillatory homeostats appear to be operative also under signaling conditions.

**Figure 10:**
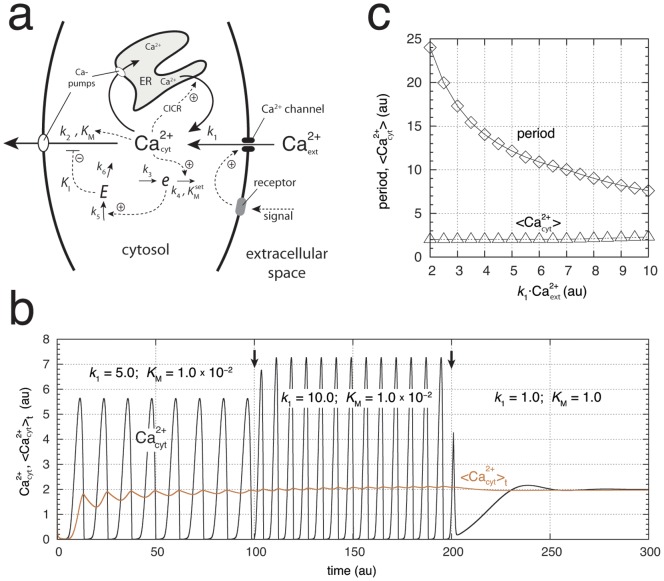
A homeostatic model of cytosolic Ca^2+^ oscillations. The model considers a stimulated non-excitable cell under stationary conditions using an extended version of outflow controller motif 6, where 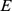 is the controller molecule. Intermediate 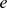 has been included to get limit-cycle oscillations. Rate constant 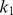 describes the total inflow of Ca^2+^ from the ER and from the extracellular space into the cytosol and reflects the strength of the stimulation. For the sake of simplicity the external Ca^2+^ concentration (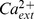) is considered to be constant (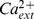 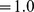). 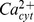 denotes cytosolic Ca^2+^ and its concentration. (a) Reaction scheme. Rate equations: 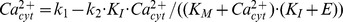; 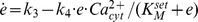; 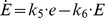. Rate constants: 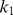, variable; 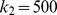; 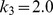; 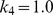; 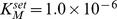; 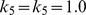; 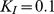. The homeostat's set-point for Ca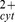 is given by 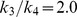. (b) 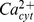 oscillations and average cytosolic Ca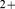 concentration, 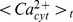, at different stimulations and as a function of time 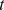. Initial concentrations: 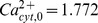, 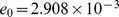; 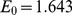. The quenching of oscillations at low 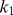 is due to an increased 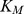 value. (c) Period length and average cytosolic Ca^2+^ concentration (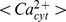) calculated after 2000 time units for different stimulation strengths (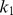 values). Same rate constants as in (b) with 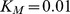. Initial concentrations for each calculated data point: 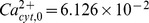, 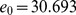; 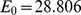.

Although a variety of mathematical models have been suggested to describe Ca^2+^ oscillations [78]–[84], none of them have so far included an explicit homeostatic regulation of cytosolic Ca^2+^. Fig. 10a shows how Ca^2+^ oscillations can be obtained based on an outflow homeostatic controller, which removes excess and toxic amounts of cytosolic Ca^2+^. The model considers a stationary situation of an activated cell, where a Ca^2+^ channel is activated by an external signal leading to the inflow of Ca^2+^ into the cytosol. The increased Ca^2+^ levels in the cytosol induce an additional inflow of Ca^2+^ from the internal Ca^2+^ store, a mechanism termed “Calcium-Induced Calcium Release” (CICR) [85]. Both inflows are lumped together and described by rate constant 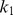. The CICR flux is maintained by pumping cytosolic Ca^2+^ into the ER and keeping the Ca^2+^ load in the ER high. It should be mentioned that the cause of the Ca^2+^ entry across the plasma membrane into the cytosol is not fully understood and different views have been expressed how this can occur [86], [87].

For the sake of simplicity, the Ca^2+^ concentration in the ER is considered to be constant and only the pumping of Ca^2+^ from the cytosol into the extracellular space is taken into account without an increased cooperativity (Hill-function) with respect to the Ca^2+^ concentration. Fig. 10b shows the oscillations of cytosolic Ca^2+^ and the homeostat's performance at different inflow rates 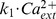 into the cytosol, which can reflect different external Ca^2+^ concentrations and/or different activation levels of the cell. As observed experimentally [74] the period of the oscillations decreases with increased external Ca^2+^ concentration or with an increased stimulation of the cell. As shown by 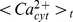 in Fig. 10b and by total 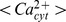 in Fig. 10c, on average, robust Ca^2+^ homeostasis is preserved at varying Ca^2+^ inflow rates. In the absence of oscillations the Ca^2+^ concentration is still kept at its homeostatic set-point (Fig. 10b).

Why Ca^2+^ oscillations? A non-oscillatory signaling mechanism by cytosolic Ca^2+^ would clearly be limited, because a homeostatic regulation of cytosolic Ca^2+^ would not allow varying Ca^2+^ levels as a function of external stimulation strengths. On the other hand, a frequency-based signaling due to an oscillatory Ca^2+^-homeostat would overcome these limitations, because homeostasis is still maintained. This has been a brief outline on how Ca^2+^ oscillations may be understood on basis of oscillatory homeostasis. More detailed studies will be needed, for example by including the homeostatic aspect in existing models in order to investigate in more detail the implications oscillatory homeostats have on the regulatory role of Ca^2+^.

#### p53 Signaling

p53 is a transcription factor with tumor suppressor properties. In more than half of all human tumors p53 is mutated and in almost all tumors p53 regulation is not functional [88]. In the presence of DNA damage and other abnormalities p53 initiates the removal of damaged cells by apoptosis. A central negative feedback component in p53 regulation is Mdm2, an ubiquitin E3 ligase, which leads to the proteasomal degradation of p53 and other tumor suppressors [89]. In the presence of DNA damage, p53 is upregulated by several mechanisms [90]–[92], and both p53 and Mdm2 have been found to oscillate [60]. An interesting feature of these oscillations is that their amplitude is highly variable, while their frequency is fairly constant [60]. The mean height of the oscillations was found to be constant [61]. It was also found that with an increased strength of DNA damaging radiation the number of cells with increased p53 cycles increased statistically [61]. Jolma *et al*. [51] used the basic negative feedback motif 5 (where 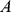 is p53 and 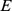 is Mdm2) and found that the influence of noise on the harmonic properties of the oscillations was able to describe the variable amplitudes and the approximately constancy of the period. Fourier analysis of the experimental data indeed showed that the p53-Mdm2 oscillations have a major harmonic component [93] supporting a quasi-harmonic character of the p53-Mdm2 oscillations. For such harmonic or quasi-harmonic oscillations our results (Figs. 2f and 5b) indicate that p53 is homeostatic regulated both in average concentration and in period length to allow to expose the system probably to an optimum amount of p53 during each cycle. Because the number of p53 cycles appear positively correlated with an increased exposure of damaging radiation, the total amount of released p53 may be related to a repair mechanism. A support along these lines comes from a recent study, which indicates that p53 oscillations lead to the recovery of DNA-damaged cells, while p53 levels kept at their peak value lead to senescence and to a permanent cell cycle arrest [94]. Thus, like for cytosolic Ca^2+^, elevated and oscillatory p53 levels seem to remain under homeostatic control in order to mediate signaling events and information which appear to be encoded in the oscillations.

#### Homeostasis of the Circadian Period

Circadian rhythms play an important role in the daily and seasonal adaptation of organisms to their environment and act as physiological clocks [8], [95], [96]. Functioning as clocks, their period is under homeostatic regulation towards a variety of environmental influences, such as changing temperature (“temperature compensation”) or food supply (“nutritional compensation”). Circadian rhythms participate in the homeostatic control of a variety of physiological variables, such as body temperature, potassium content, hormone levels, as well as sleep [1], [8], [95], [96]. As an example, potassium homeostasis in our bodies is under a circadian control, where potassium ion is daily excreted with peak values at the middle of the day [1].

One of the questions still under discussion is how the circadian period 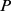 is kept under homeostatic control as for example seen in temperature compensation. In the antagonistic balance approach [97] the variation of the period 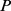 with respect to temperature 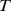, expressed as 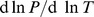, is given as the sum of the control coefficients [36] 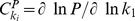 multiplied with the 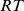-scaled activation energies 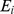 (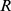 is the gas constant): 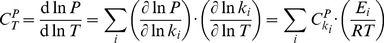
(11)The sum runs over all temperature-dependent processes 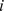 with rate constants 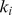, where the temperature dependence of the rate constants is expressed in terms of the Arrhenius equation 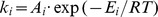 [98]. 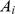 is the so-called pre-exponential factor and can, to a first approximation, be treated as temperature-independent. Eq. 11 applies to any kinetic model as long as the temperature dependence of the individual reactions are formulated in terms of the Arrhenius law.

The condition for temperature compensation is obtained by setting Eq. 11 to zero. Because in oscillatory systems the 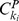
's have generally positive and negative values, there is a large set of balancing 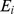 combinations which can lead to temperature compensation. The various combinations can be considered to arise by evolutionary selective processes acting on the activation energies [99]. Because the temperature homeostasis of circadian rhythms involves a compensatory mechanism [100], which needs to be distinguished from temperature-independence where all 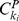
's are zero, temperature compensation implies that there is a certain set of non-zero control coefficients with associated activation energies which (under ideal conditions) will satisfy the balancing condition 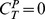 within a certain temperature range.

The argument has been made that the balancing condition 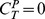 should be non-robust and should therefore not match the many examples where mutations have no influence on the circadian period [101]. However, it should be noted that Eq. 11 is *model-independent* and provides a general description how the period of an oscillator will depend on temperature in terms of the individual reactions defined by the 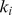
's. Robustness, on the other hand, is a property of the actual oscillator model, where the number of zero 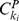
's can be taken as a measure for robustness. For the frequency controlled oscillators described earlier, there are certain regions in parameter space such as the shaded region in Fig. 7a, for which the oscillator's period is independent towards variations of those 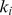
's which lie within this region. As a result, frequency controlled oscillators will show an increased robustness against environmental factors that affect rate constants, such as pH, salinity, or temperature [43], [98] and therefore appear to be candidates for modeling temperature compensation.

We feel that the here shown possibilities how robust concentration and period homeostasis can be achieved provide a new handle how the negative (and positive) feedback regulations in circadian pacemakers [102] can be approached. The incorporation of these principles into models of circadian rhythms may provide further insights how temperature compensation is achieved and how circadian rhythms participate in the homeostatic regulation of organisms [1], [103].

## Materials and Methods

Computations were performed by using Matlab/Simulink (mathwork.com) and the Fortran subroutine LSODE [104]. Plots were generated with gnuplot (www.gnuplot.info)/Matlab. To make notations simpler, concentrations of compounds are denoted by compound names without square brackets. All concentrations, time units, and rate constants are given in arbitrary units (au).

## Supporting Information

File S1
**(with Figs. S1–S14 and Eqs. S1–S57), contains derivation of the set-point under oscillatory conditions, construction of the** ***H*** **-function in conservative systems, the harmonic approximation of the frequency in conservative controllers, quenching of quasi-harmonic oscillations, and an alternative example of** 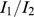 **feedback leading to robust frequency control in a motif 2 based limit-cycle oscillator.**
(PDF)
