# Supplementary material for: Robust Concentration and Frequency Control in Oscillatory Homeostats
Source: PLoS One. 2014 Sep 19;9(9):e107766. doi: 10.1371/journal.pone.0107766 (PMC4169565; doi:10.1371/journal.pone.0107766)
Supplement: File S1 — (with Figs. S1–S14 and Eqs. S1–S57), contains derivation of the set-point under oscillatory conditions, construction of the H -function in conservative systems, the harmonic approximation of the frequency in conservative controllers, quenching of quasi-harmonic oscillations, and an alternative example of feedback leading to robust frequency control in a motif 2 based limit-cycle oscillator. (PDF) [file pone.0107766.s001.pdf]

Supporting Material, File S1

Robust Concentration and Frequency Control in Oscillatory Homeostats

K. Thorsen[1], O. Agafonov[2], C. H. Selstø[2], I. W. Jolma[2], X. Y. Ni[2], T. Drengstig[1], and P. Ruoff[2*]

[1]Department of Electrical Engineering and Computer Science,
[2]Centre for Organelle Research
University of Stavanger, Stavanger, Norway

[*]Corresponding author. Address: Centre for Organelle Research, University of Stavanger, N-4036 Stavanger, Norway, Tel.: (47) 5183-1887, Fax: (47) 5183-1750, E-mail: peter.ruoff@uis.no

Set-point of Controlled Variable A under Oscillatory Conditions

A-Activating Controller Motifs

The A-activating motifs are 1, 2, 5, and 6 (Fig. 1, main paper). As an example we use the harmonic oscillator described in Fig. 2d. The rate equation for E is given as:

$$\dot{E} = k_4 \cdot A - \frac{V_{max}^{E_{set}} \cdot E}{K_M^{E_{set}} + E} \tag{S1}$$

Integral control is introduced by zero-order kinetics when $K_M^{E_{set}}$ becomes negligible in comparison to E (1, 2). Under oscillatory conditions the set-point in the average concentration of A, $<A>_c$, is obtained by using the following condition

$$<\dot{E}>_c \; = \oint_c \dot{E}\, dt = \frac{1}{P}\int_0^P \dot{E}\, dt = 0 \tag{S2}$$

where integration occurs along one orbit/cycle of stable oscillations with period P. By inserting the expression of $\dot{E}$ (Eq. S1) into Eq. S2 we get

$$<\dot{E}>_c \; = k_4 \cdot <A>_c - V_{max}^{E_{set}} \left\langle \frac{E}{K_M^{E_{set}} + E} \right\rangle_c = 0 \tag{S3}$$

Using ideal zero-order condition, $K_M^{E_{set}} \rightarrow 0$, we have $<E/(K_M^{E_{set}}+E)>_c \rightarrow 1$, i.e.,

$$\left\langle \frac{E}{K_M^{E_{set}}+E} \right\rangle_c = \lim_{K_M \rightarrow 0} \left\{ \frac{1}{P}\int_o^P \left(\frac{E}{K_M+E} \right) dt \right\} = \frac{1}{P}\int_o^P \lim_{K_M \rightarrow 0} \left\{ \frac{E}{K_M+E} \right\} dt$$

$$= \frac{1}{P}\int_o^P 1\, dt = 1 \tag{S4}$$

Inserting this result into Eq. S3, we get

$$<A>_c = \frac{V_{max}^{E_{set}}}{k_4} = <A>_{set} \tag{S5}$$

Note that $<A>_c$ is identical with the set-point of A when the system is non-oscillatory. This is shown in Fig. 3b and Fig. 5d for limit-cycle oscillators based on motif 2 and 5, respectively.

A-Inhibiting Controller Motifs

The A-inhibiting controller motifs are 3, 4, 7 and 8. As an example we use controller motif 3.

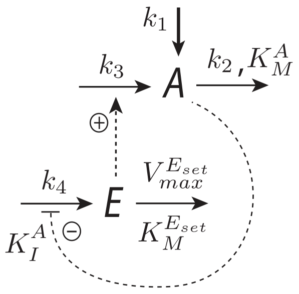

Figure S1. Motif 3.

The rate equation for E is given as:

$$\dot{E} = \frac{k_4 \cdot K_I^A}{K_I^A + A} - \frac{V_{max}^{E_{set}} \cdot E}{K_M^{E_{set}} + E} \tag{S6}$$

Considering zero-order conditions in the Michaelis-Menten removal of E, the condition $<\dot{E}>_c$=0 gives:

$$<\dot{E}>_c \; = k_4 \cdot K_I^A \left\langle \frac{1}{K_I^A + A} \right\rangle_c - V_{max}^{E_{set}} = 0 \tag{S7}$$

$$\Rightarrow \quad \left\langle \frac{1}{K_I^A + A} \right\rangle_c \overset{\text{def}}{=} \frac{1}{P} \int_o^P \frac{1}{K_I^A + A} \, dt = \frac{V_{max}^{E_{set}}}{k_4 \cdot K_I^A} \tag{S8}$$

where P is the period of the oscillator and $V_{max}^{E_{set}}/k_4 \cdot K_I^A$ is the homeostatic conserved property. In case the system becomes non-oscillatory the homeostatic set-point of A, A_{set}, is given as (2):

$$\frac{1}{K_I^A + A_{set}} = \frac{V_{max}^{E_{set}}}{k_4 \cdot K_I^A} \quad \Rightarrow \quad A_{set} = \frac{k_4 \cdot K_I^A}{V_{max}^{E_{set}}} - K_I^A \tag{S9}$$

Conservative Oscillator Types and Construction of their H-functions

We illustrate here the construction of the H-functions of the four different conservative oscillator types that can be constructed by using motif 2.

Oscillator with both A and E Removals Being Zero-Order

Fig. 2a in the main paper shows the reaction scheme and rate equations for this case. For the sake of simplicity we assume that k_1=0. The H-function obeys the following equations, which are analogous to the Hamilton-Jacobi equations (3)

$$\frac{\partial H}{\partial E} = -\dot{A}; \quad \frac{\partial H}{\partial A} = \dot{E} \tag{S10}$$

The H-function is constructed by integrating $\dot{A}$ and $\dot{E}$, i.e.,

$$H = -\int \dot{A}\,\mathrm{d}E + \int \dot{E}\,\mathrm{d}A \tag{S11}$$

Applying the zero-order conditions with respect to the removal kinetics of A and E, we get

$$H = -\int \left(-k_2 + \frac{k_3 \cdot K_I^E}{K_I^E + E}\right) \mathrm{d}E + \int \left(k_4 \cdot A - V_{max}^{E_{set}}\right) \mathrm{d}A \tag{S12}$$

which leads to the final expression of H (see also Fig. S2)

$$H = k_2 \cdot E - k_3 \cdot K_I^E \cdot \ln(K_I^E + E) + \tfrac{1}{2} k_4 \cdot A^2 - V_{max}^{E_{set}} \cdot A \tag{S13}$$

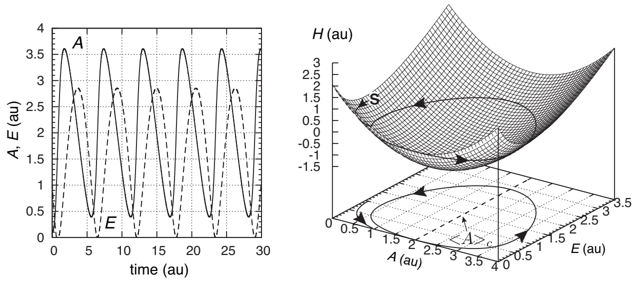

Figure S2. Left panel: conservative oscillations in A and E using motif 2 with rate constant as given in Fig. 2b (t<50, k_2=1). Initial concentrations A_0=0.1 and E_0=1.0. Right panel: H-function (Eq. S13) showing the oscillations of the left panel as curves in A-E phase space and on the surface of H. Dashed line indicates the oscillator's set-point $<A>_c$=2.0.

Oscillator with Autocatalysis in A and E and First-Order Removals

In the case the degradation of E is first order, integral control in A can be implemented by first-order autocatalysis in E (3). To keep the system conservative with first-order degradation in A, the formation in A needs to be first-order autocatalytic as indicated in Fig. S3 and shown by the rate equations.

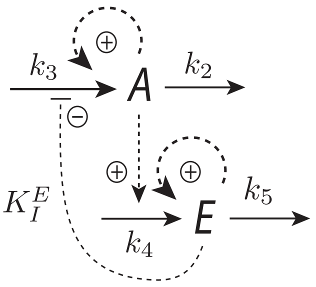

Figure S3. Motif 2 with autocatalysis and first-order degradations in A and E.

The rate equations are:

$$\dot{A} = \frac{k_3 \cdot K_I^E}{K_I^E + E} \cdot A - k_2 \cdot A \tag{S14}$$

$$\dot{E} = k_4 \cdot A \cdot E - k_5 \cdot E \tag{S15}$$

Introducing the variables $\xi = \ln A$ and $\eta = \ln E$, the rate equations can be transformed to:

$$\dot{\xi} = \frac{\dot{A}}{A} = \frac{k_3 \cdot K_I^E}{K_I^E + E} - k_2 = \frac{k_3 \cdot K_I^E}{K_I^E + e^{\eta}} - k_2 \tag{S16}$$

$$\dot{\eta} = \frac{\dot{E}}{E} = k_4 \cdot A - k_5 = k_4 \cdot e^{\xi} - k_5 \tag{S17}$$

By expressing E and A in Eqs. S16 and S17 in term of ξ and η, the function

$$H(\xi, \eta) = \int \dot{\xi} \, \mathrm{d}\eta - \int \dot{\eta} \, \mathrm{d}\xi \tag{S18}$$

describes the kinetics of this conservative system and, after integration, is given by

$$H(\xi,\eta) = (k_3 - k_2)\cdot\eta - k_3\cdot\ln(K_i^E + e^{\eta}) - k_4\cdot e^{\xi} + k_5\cdot\xi \tag{S19}$$

By using $\xi = \ln A$ and $\eta = \ln E$, H can be expressed in terms of A and E, i.e,

$$H(A,E) = (k_3 - k_2)\cdot\ln E - k_3\cdot\ln(K_i^E + E) - k_4\cdot A + k_5\cdot\ln A \tag{S20}$$

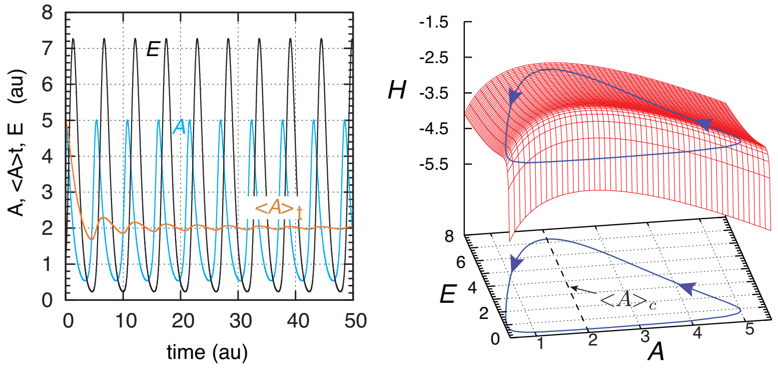

Figure S4. Left panel: conservative oscillations in A, E, and $<A>_t$ as a function of time using motif 2 when both A and E are formed autocatalytically and degraded by first-order reactions (eqs. S14 and S15). Rate constants: K_I^E=0.1, k_2=1.0, k_3=10.0, k_4=1.0, k_5=2.0 Initial concentrations A_0=5.0 and E_0=1.0. Right panel: H-function (Eq. S20) showing the oscillations of the left panel as curves in A-E phase space and on the surface of H. Dashed line indicates the oscillator's set-point $<A>_c$=2.0.

Oscillator with Autocatalysis in A and Zero-order Removal of E

The scheme of this conservative oscillator is given in Fig. S5. The rate equations are:

$$\dot{A} = \frac{k_3\cdot K_I^E}{K_I^E + E}\cdot A - k_2\cdot A \tag{S21}$$

$$\dot{E} = k_4\cdot A - \frac{V_{max}^{E_{set}}\cdot E}{K_M^{E_{set}} + E} \tag{S22}$$

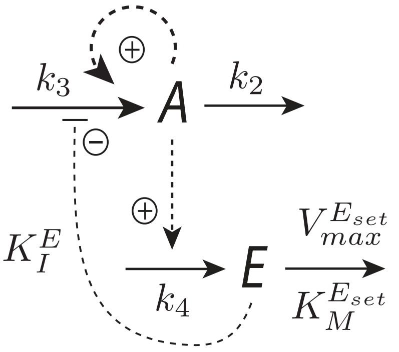

Figure S5. Motif 2 with autocatalysis and first-order degradation in A and and zero-order removal of E.

The H-function is given by the following integral:

$$H(\xi, E) = -\int \dot{\xi}\, \mathrm{d}E + \int \dot{E}\, \mathrm{d}\xi \tag{S23}$$

$$= -k_3 k_6 \ln(k_6{+}E) + k_2 E + k_4 e^{\xi} - k_5 \xi \tag{S24}$$

where

$$\dot{\xi} = \frac{\dot{A}}{A} = \frac{k_3{\cdot}K_I^E}{K_I^E + E} - k_2 \tag{S25}$$

Inserting the expression $\xi{=}\ln(A)$ into Eq. S24 gives the final form of $H(A, E)$:

$$H(A, E) = -k_3{\cdot}k_6{\cdot} \ln(k_6{+}E) + k_2{\cdot}E + k_4{\cdot}A - k_5{\cdot} \ln(A) \tag{S26}$$

Fig. S6 shows the numerically calculated oscillations and the constructed H-function describing these oscillations in phase-space.

Oscillator with Zero-order Removal of A and Autocatalysis in E

The reaction scheme of this conservative oscillator is given in Fig. S7.

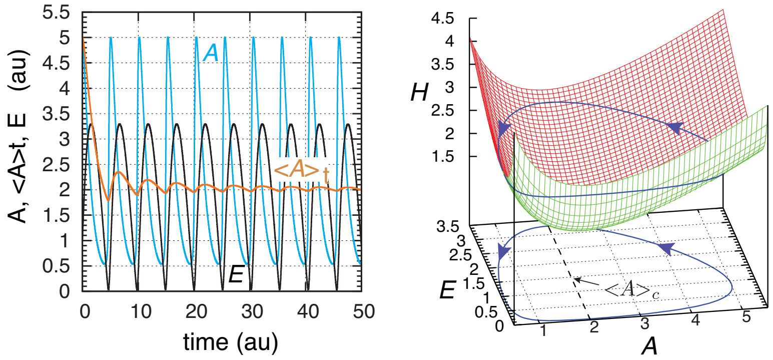

Figure S6. Left panel: conservative oscillations in A, E, and $<A>_t$ as a function of time using motif 2 (Fig. S5) and rate eqns. S21 and S22. Rate constants: K_I^E=0.1, k_2=1.0, k_3=10.0, k_4=1.0, $V_{max}^{E_{set}}$=2.0, and $k_M^{E_{set}}=1\times10^{-6}$. Initial concentrations A_0=5.0 and E_0=1.0. Right panel: H-function (Eq. S26) showing the oscillations of the left panel as curves in A-E phase space and on the surface of H. Dashed line indicates the oscillator's set-point $<A>_c$=2.0.

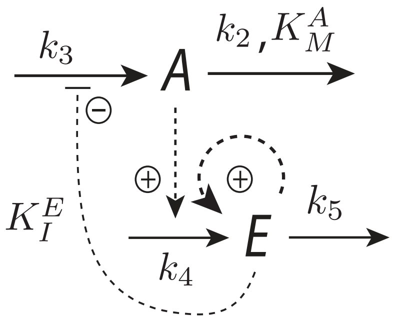

Figure S7. Motif 2 with autocatalysis and first-order degradation in A and zero-order removal in E.

The rate equations are:

$$\dot{A} = \frac{k_3 \cdot K_I^E}{K_I^E + E} - \frac{k_2 \cdot A}{K_M^A + A} \tag{S27}$$

$$\dot{E} = k_4 \cdot A \cdot E - k_5 \cdot E \tag{S28}$$

The H-function is given by the integral:

$$H(A,\eta) = -\int \dot{A}\,\mathrm{d}\eta + \int \dot{\eta}\,\mathrm{d}A \tag{S29}$$

where

$$\eta = \ln E \quad \text{and} \quad \dot{\eta} = \frac{\dot{E}}{E} = k_4{\cdot}A - k_5 \tag{S30}$$

$$\Rightarrow \quad H(A,\eta) = -\int \left(\frac{k_3 K_I^E}{K_I^E{+}e^{\eta}} {-} k_2\right) \mathrm{d}\eta + \int (k_4 A - k_5)\,\mathrm{d}A \tag{S31}$$

$$= -k_3 K_I^E \int \frac{\mathrm{d}\eta}{K_I^E{+}e^{\eta}} {+} k_2\eta + \tfrac{1}{2}k_4{\cdot}A^2 - k_5 A \tag{S32}$$

$$= -k_3\eta {+} k_3 \ln(K_I^E{+}e^{\eta}) {+} k_2\eta + \tfrac{1}{2}k_4{\cdot}A^2 - k_5 A \tag{S33}$$

where

$$\int \frac{\mathrm{d}\eta}{K_I^E{+}e^{\eta}} = \frac{1}{K_I^E}\left(\eta - \ln(K_I^E{+}e^{\eta})\right) \tag{S34}$$

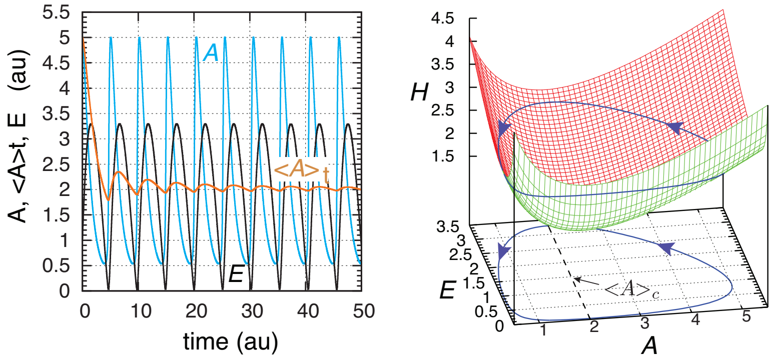

Figure S8. Left panel: conservative oscillations in A, E, and $<A>_t$ as a function of time using motif 2 (Fig. S7) and rate eqns. S27 and S28). Rate constants: K_I^E=0.1, k_2=1.0, k_3=10.0, k_4=1.0, k_5=2.0, and K_M^A=1×10^{-6}. Initial concentrations A_0=3.0232 and E_0=10.2342. Right panel: H-function (Eq. S35) showing the oscillations of the left panel as curve in A-E phase space and on the surface of H. The curve on the H-surface is located at H=0.9464 (calculated from the initial concentration A_0 and E_0). Dashed line indicates the homeostats's set-point $<A>_c$=2.0.

Inserting $\eta = \ln(E)$ into Eq.S33 leads to

$$H(A,E) = -k_3 \ln(E) + k_3 \ln(K_I^E + E) + k_2 \ln(E) + \tfrac{1}{2} k_4 \cdot A^2 - k_5 A \qquad \text{(S35)}$$

Fig. S8 shows the numerically calculated oscillations and the constructed H-function.

Harmonic Approximation of Frequency in Conservative Oscillatory Controllers

The harmonic approximation of the frequency in conservative controllers provides insights why oscillatory controllers based on even-numbered motifs (Fig. 1) increase their frequency upon increased perturbation strengths. As an example we show how the harmonic approximation of the frequency can be obtained for the conservative oscillatory controller based on motif 2 (Fig. 2a). We assume zero-order removal in A and E and $k_1 = 0$. The rate equations read then:

$$\dot{A} = \frac{k_3 \cdot K_I^E}{K_I^E + E} - k_2 \qquad \text{(S36)}$$

$$\dot{E} = k_4 \cdot A - V_{max}^{E_{set}} \qquad \text{(S37)}$$

Taking the second time derivative of Eq. S36 gives:

$$\ddot{A} = -\frac{k_3 \cdot K_I^E}{(K_I^E + E)^2} \cdot \dot{E} = -\frac{k_3 \cdot K_I^E}{(K_I^E + E)^2} (k_4 A - V_{max}^{E_{set}}) \qquad \text{(S38)}$$

Eq. S38 can be rearranged into the following form:

$$\frac{\ddot{A}}{\frac{k_3 \cdot k_4 \cdot K_I^E}{(K_I^E + E)^2}} + A = \frac{V_{max}^{E_{set}}}{k_4} = A_{ss} = \langle A \rangle_{set} \qquad \text{(S39)}$$

When E in Eq. S39 is replaced by E_{ss} we get the equation of a harmonic oscillator, i.e., $\ddot{A}/\omega^2 + A$=constant, with frequency ω given as

$$\omega = \sqrt{\frac{k_3 \cdot k_4 \cdot K_I^E}{(K_I^E + E_{ss})^2}} \qquad \text{(S40)}$$

and which approximately describes the frequency of the conservative oscillator (Eq. S39). A corresponding second-order differential equation can be derived for E:

$$\frac{\ddot{E}}{\frac{k_3 \cdot k_4 \cdot K_I^E}{(K_I^E+E)(K_I^E+E_{ss})}} + E = \frac{k_3 \cdot K_I^E}{k_2} - K_I^E = E_{ss} \tag{S41}$$

A_{ss} and E_{ss} denote the steady state concentrations when $\dot{A}$=0 and $\dot{E}$=0. When replacing E by E_{ss} in Eq. S41 the same harmonic frequency approximation as described by Eq. S40 is obtained. Similar expressions are found for the other E-inhibiting oscillatory controllers. Because the level of E decreases with increasing perturbation strength, Eq. S40 indicates that the E-inhibiting controllers will increase their frequency when perturbations are increased as shown in Fig. 3 for the motif-2-based controller.

For the conservative oscillators based on motifs 4 and 8, i.e., when both A and E are inhibiting, the harmonic oscillator approximations are:

$$\frac{\ddot{A}}{\frac{k_i \cdot k_j \cdot K_I^E \cdot K_I^A}{(K_I^E+E_{ss})^2(K_I^A+A_{ss})^2}} + A = A_{ss} \tag{S42}$$

$$\frac{\ddot{E}}{\frac{k_i \cdot k_j \cdot K_I^E \cdot K_I^A}{(K_I^E+E_{ss})^2(K_I^A+A_{ss})^2}} + E = E_{ss} \tag{S43}$$

where k_i and k_j denote rate constants of the reactions which are inhibited by A and E.

For conservative oscillators based on motifs 1 and 5, the frequency is not dependent on either A or E giving harmonic oscillators (see next section).

Harmonic Oscillations (Inflow Controller Motif 1)

Fig. S9 shows a two-component representation of motif 1. When $K_M^A \ll A$ and $K_M^{E_{set}} \ll E$ the system becomes an harmonic oscillator with set point $<A>_c = k_4/V_{max}^{E_{set}}$.

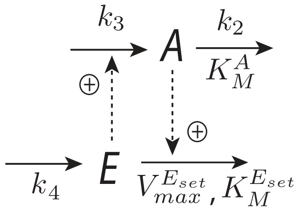

Figure S9. Motif 1. The two-component system shows harmonic oscillations when $K_M^A \ll A$ and $K_M^{E_{set}} \ll E$, i.e. removal of A and E follow zero-order kinetics.

We consider the rate equations:

$$\dot{A} = k_3 \cdot E - \frac{k_2 \cdot A}{K_M^A + A} \tag{S44}$$

$$\dot{E} = k_4 - \frac{V_{max}^{E_{set}} \cdot E}{K_M^{E_{set}} + E} \cdot A \tag{S45}$$

In case of zero-order conditions in the removal of A and E they reduce to:

$$\dot{A} = k_3 \cdot E - k_2 \tag{S46}$$

$$\dot{E} = k_4 - V_{max}^{E_{set}} \cdot A \tag{S47}$$

Taking the second time derivative of Eq. S46 and inserting the expression of $\dot{E}$ into it, leads to:

$$\ddot{A} = k_3 \cdot \dot{E} = k_3 \cdot k_4 - k_3 \cdot V_{max}^{E_{set}} \cdot A \tag{S48}$$

Dividing Eq. S48 by $k_3 \cdot V_{max}^{E_{set}}$ gives the equation of a harmonic oscillator around the set-point $<A>_c$:

$$\frac{\ddot{A}}{\omega^2} + A = \frac{k_4}{V_{max}^{E_{set}}} = <A>_c \tag{S49}$$

where $A(t)$ is given as:

$$A(t) = A_{amp} \sin(\omega \cdot t + \phi) + <A>_c \tag{S50}$$

A_{amp} denotes the A-amplitude of the oscillations, ω is the frequency, and ϕ is a phase angle.

Quenching of Oscillations in Quasi-Conservative Systems

First-Order Degradation Restrains Oscillations

The amount of uncontrolled first-order degradation of A has a major influence on the size of the parameter space for the extended motif 5 (Figs. 4a and 5d in main paper) in which sustained oscillations are observed.Our results show that first-order degradation has the ability to quench the oscillations and does so for a large range of parameters. Fig. S10 shows how an increasing first-order rate constant in the removal of A reduces the oscillatory behavior in the k_1 (uncontrolled inflow of A)-k_5 (conversion of precursor e into E) parameter space. The parameter space in which sustained oscillations are observed shrinks markedly when when the first-order degradation rate constant k_3 is increased by one order of magnitude, i.e. from 0.01 to 0.1 (Fig. S10, panel c)

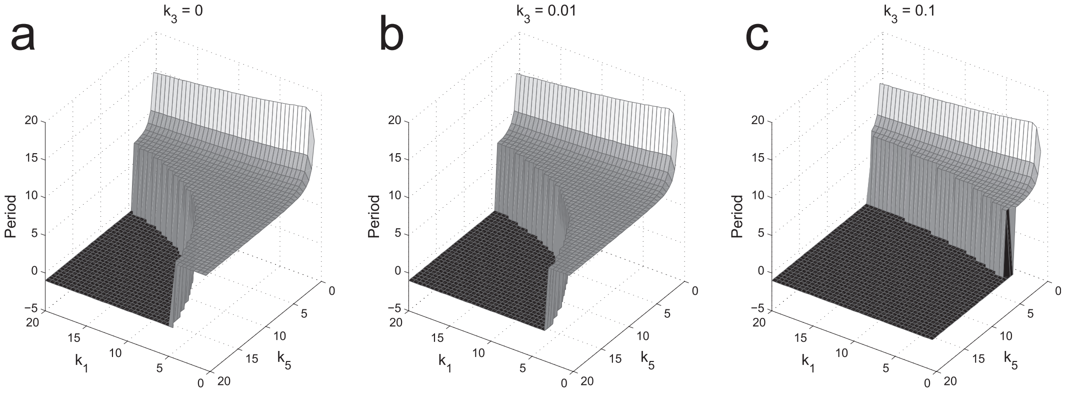

Figure S10. Period of oscillations for varying k_1 and k_5 values using the limit-cycle verion of motif 5 described in the main paper (Fig. 4a). The period is set to -1 when there are no oscillations (black area). Panels (a), (b), and (c) show the results for three different values for the first-order A-removing rate constant k_3. The parameter values used are: k_2=0.5, k_4=0.7, $V_{max}^{E_{set}}$=0.5, K_M^A=4 $\times 10^{-3}$, and $K_M^{E_{set}}$=1 $\times 10^{-2}$.

Fig. S10 also shows the propagation towards quasi-harmonic kinetics. With increasing k_5 values the periods approaches the harmonic values of $2\pi/\sqrt{k_2 k_4}$. When the conversion from e to E is fast (high k_5), the motif gives quasi-

harmonic oscillatory period homeostasis as discussed in the main paper. A very fast conversion from e to E does however lead to a non-oscillatory homeostasis in A, i.e., the range of k_1 values that give oscillations shrinks with increasing k_5.

Zero-Order Degradation Facilitates Oscillations

How close the controlled degradation of A is to a perfect zero-order degradation is another factor that influences the size of the oscillatory regime. With a controlled degradation of A by the compensatory flux j_{comp}

$$j_{comp} = \frac{k_2 \cdot A \cdot E}{K_M^A + A} \tag{S51}$$

K_M^A becomes an indicator of how close the degradation is to perfect zero-order, i.e. when $K_M^A \to 0$. Fig. S11 shows the size of the parameter space in which one observes oscillatory behavior in the extended motif 5 (Fig 4a in the main paper) for three different values of K_M^A. The uncontrolled first-order degradation rate constant k_3 is 1×10^{-2} in all cases.

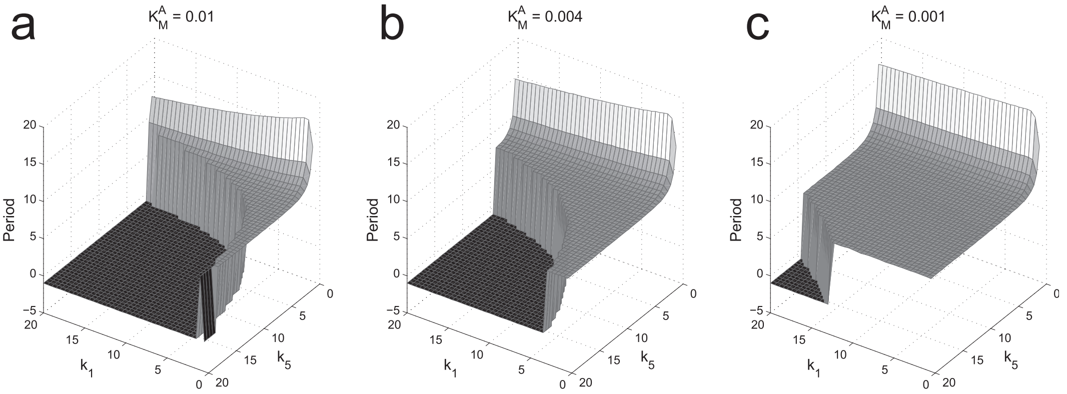

Figure S11. Period of oscillations for varying k_1 and k_5 values using the limit-cycle verion of motif 5 described in the main paper (Fig. 4a). The period is set to -1 when there are no oscillations (black area). Panels (a), (b), and (c) show the results for three different values of K_M^A. The parameter values used are: k_2=0.5, k_3=1×10^{-2}, k_4=0.7, $V_{max}^{E_{set}}$=0.5, and $K_M^{E_{set}}$=1×10^{-2}.

Robust Frequency Control With Inflow Controller Motif 2 and Alternative I_1/I_2 Feedback

In the main manuscript the feedbacks from I_1 and I_2 were applied on intermediate a (Fig. 7) or were "mixed", i.e. were applied to both "a" and "A" (Fig. 8). In the following we show a model where the feedbacks from I_1/I_2 are returned to A only. The scheme is given in Fig. S12.

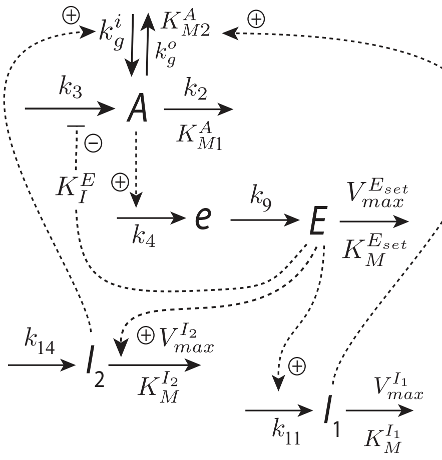

Figure S12. Alternative I_1/I_2 feedback arrangement in a motif-2-based oscillator. The feedbacks from I_1 and I_2 act on A only.

The rate equations are:

$$\dot{A} = \frac{k_3 \cdot K_I^E}{K_I^E + E} + k_g^i \cdot I_2 - \left(\frac{k_g^o \cdot A}{K_{M2}^A + A} \right) \cdot I_1 - \frac{k_2 \cdot A}{K_{M1}^A + A} \tag{S52}$$

$$\dot{e} = k_4 \cdot A - k_9 \cdot e \tag{S53}$$

$$\dot{E} = k_9 \cdot e - \frac{V_{max}^{E_{set}} \cdot E}{K_M^{E_{set}} + E} \tag{S54}$$

$$\dot{I}_1 = k_{11} \cdot E - \frac{V_{max}^{I_1} \cdot I_1}{K_M^{I_1} + I_1} \tag{S55}$$

$$\dot{I}_2 = k_{14} - \left(\frac{V_{max}^{I_2} \cdot I_2}{K_M^{I_2} + I_2} \right) \cdot E \tag{S56}$$

The controller molecules (manipulated variables) E, I_1, and I_2 define the set-points $<A>_{set}$, $<E>_{set}^{I_1}$, and $<E>_{set}^{I_2}$, respectively, which are given by

$$<A>_{set} = \frac{V_{max}^{E_{set}}}{k_4}; \quad <E>_{set}^{I_1} = \frac{V_{max}^{I_1}}{k_{11}}; \quad <E>_{set}^{I_2} = \frac{k_{14}}{V_{max}^{I_2}} \qquad \text{(S57)}$$

Fig. S13 shows the oscillator's behavior, i.e., $<A>$, $<E>$, and the frequency as a function of the perturbation k_2 when $<A>_{set}$=2.0, $<E>_{set}^{I_1} = 5.0$, and $<E>_{set}^{I_2} = 2.0$. Due to the two set-points $<E>_{set}^{I_1}$ and $<E>_{set}^{I_2}$ the frequency has a corresponding homeostatic regulation at two frequencies. Note that, although $<E>$ changes between different set-points when k_2 is changed, $<A>$ is kept at its homeostatic set-point $<A>_{set}$=2.0.

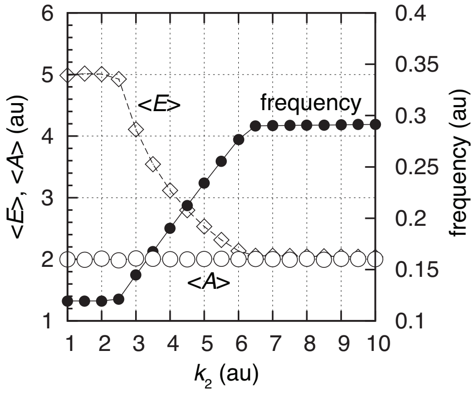

Figure S13. $<A>$, $<E>$, and the frequency as a function of the perturbation strength k_2. Rate constant values: k_3=100.0, k_4=1.0, K_I^E=0.1, $V_{max}^{E_{set}}$=2.0, $K_M^{E_{set}}=1\times10^{-6}$, $K_{M1}^A=1\times10^{-6}$, k_9=20.0, k_{11}=1.0, $V_{max}^{I_1}$=5.0, $K_M^{I_1}=1\times10^{-6}$, k_{14}=2.0, $V_{max}^{I_2}$=1.0, $K_M^{I_2}=1\times10^{-6}$, $k_g^o=1\times10^{-2}$, $K_{M2}^A=1\times10^{-6}$, $k_g^i=1\times10^{-2}$. Initial concentrations (the same for each k_2 value): A_0=0.6677, E_0=1.0536, $e_0=2.5827\times10^{-2}$, $I_{1_0}=1.1614\times10^{-3}$, $I_{2_0}=7.5008\times10^2$. The values of $<A>$, $<E>$, and the frequency were determined after 1500 time units.

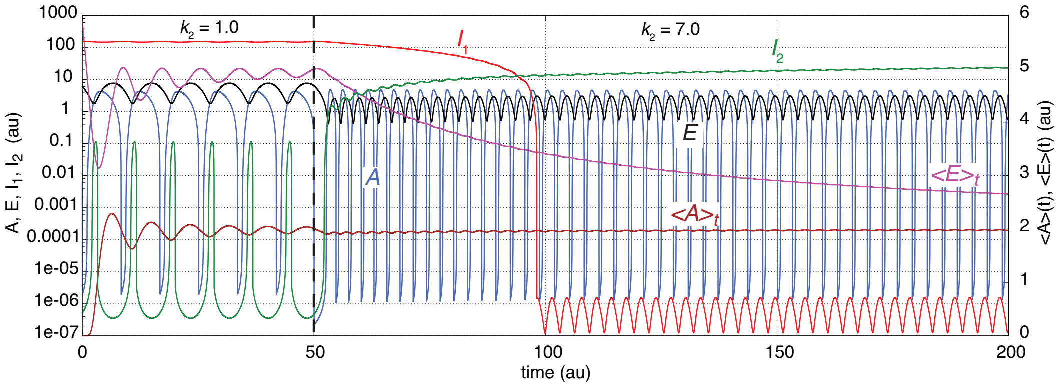

Figure S14. Time plot of the system from Fig. S13 at low and high k_2 values. At k_2=1.0 controller I_1 dominates and removes A to achieve the set-point $<E>_{set}^{I_1}$=5.0. When k_2 is high (7.0) controller I_2 is up-regulated and adds A such that $<E>$ homeostasis at $<E>_{set}^{I_2}$=2.0 is obtained. A_0=6.1923×10^{-3}, E_0=5.9023, e_0=2.5385×10^{-3}, I_{1_0}=153.26, I_{2_0}=5.1252×10^{-7}.

Fig. S14 shows the oscillations and the I_1/I_2 regulation of the system when k_2 is changed from a relative low value (k_2=1.0) to a relative high value (k_2=7.0) at time t=50.0 (dashed line). At low k_2 values controller I_1 is dominant and removes A such that this controller's set-point in $<E>$ is maintained. At high k_2 values I_2 is up-regulated and I_1 downregulated. I_2 now adds A to the system in order to keep the $<E>$ level at the set-point determined by controller I_2.

References

[1] Ni, X. Y.; Drengstig, T.; Ruoff, P. *Biophys J* **2009**, *97*, 1244–53.

[2] Drengstig, T.; Jolma, I. W.; Ni, X. Y.; Thorsen, K. G.; Xu, X. M.; Ruoff, P. *Biophys J* **2012**, *103*, 2000–10.

[3] Drengstig, T.; Ni, X. Y.; Thorsen, K.; Jolma, I. W.; Ruoff, P. *J Phys Chem B* **2012**, *116*, 5355–5363.
